# Epigenetic Age Monitoring in Professional Soccer Players for Tracking Recovery and the Effects of Strenuous Exercise

**DOI:** 10.1111/acel.70182

**Published:** 2025-07-28

**Authors:** Robert T. Brooke, Thomas Kocher, Roland Zauner, Juozas Gordevicius, Milda Milčiūtė, Marc Nowakowski, Christian Haser, Thomas Blobel, Johanna Sieland, Daniel Langhoff, Winfried Banzer, Steve Horvath, Florian Pfab

**Affiliations:** ^1^ Epigenetic Clock Development Foundation Torrance California USA; ^2^ EB House Austria, Research Program for Molecular Therapy of Genodermatoses, Department of Dermatology and Allergology University Hospital of the Paracelsus Medical University Salzburg Salzburg Austria; ^3^ DNAthlete Austria GmbH Salzburg Austria; ^4^ Eintracht Frankfurt Fußball AG Frankfurt am Main Germany; ^5^ Eurofins Genomics Europe Aarhus Denmark; ^6^ Division of Preventive and Sports Medicine, Institute of Occupational, Social and Environmental Medicine Goethe University Frankfurt Frankfurt am Main Germany; ^7^ Altos Labs Cambridge UK; ^8^ DNAthlete AG Schaan Liechtenstein; ^9^ Technische Universitaet Munich Munich Germany; ^10^ Brighton & Hove Albion Football Club Brigthon UK

**Keywords:** C‐reactive protein, exercise, FitAge, GrimAge2, soccer, sports injury

## Abstract

Elite sports have become increasingly professionalized and personalized, with soccer players facing a high number of games per season. This trend presents significant challenges in optimizing training for peak performance and requires rigorous monitoring of athletes to prevent overload and reduce injury risks. The emerging field of epigenetic clocks offers promising new pathways for developing useful biomarkers that enhance training management. This study investigates the effects of intense physical activity on epigenetic age markers in professional soccer players across multiple games and during a championship season. We analyzed DNA methylation data from saliva samples collected before and after physical activity. Vigorous physical activity was found to rejuvenate epigenetic clocks, with significant decreases in DNAmGrimAge2 and DNAmFitAge observed immediately after games. Among player subgroups, midfielders exhibited the most substantial epigenetic rejuvenation effect following games. Additionally, the study suggests a potential link between DNA methylation patterns and injury occurrence. Overall, our study suggests that DNA methylation‐based biomarkers may have applications in monitoring athlete performance and managing physical stress.

## Graphical abstract

Strenuous exercise affects biological aging in professional soccer players. DNA methylation markers analyzed from saliva samples showed epigenetic rejuvenation of the players after soccer games. Furthermore, analyses within the early recovery phase of athletes suggest a potential link between epigenetic markers and injury risk. Our study highlights the potential value of epigenetic clock analyses to predict and prevent injuries by adjusting training settings for elite athletes.

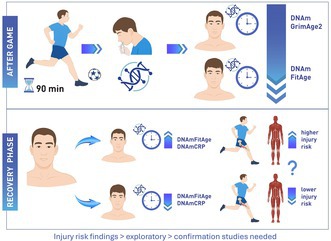

## 1 Introduction

Elite sports have experienced an enormous surge in professionalization and personalization of competition, training, prevention, and recovery management. The demands on athletes have increased owing to the evolving nature of sports and tight competition schedules, with elite soccer players playing up to 75 games per season. Besides tight time schedules and extensive travel, soccer has undergone a significant evolution in terms of the dynamic nature of the game, with increased running distances, number of runs, number of sprints, and high‐speed actions (Barnes et al. 2014; Wallace and Norton 2014; Haller et al. 2023). In recent decades, a variety of performance parameters have been established and considered to support the decision‐making processes for coaches and physicians with regard to acute load and recovery management. External load can be expressed and monitored through time‐motion analysis, tracking devices, or power parameters, like covered distance or peak power output. In addition, team physicians often rely on practical, scientifically well‐researched, and rapidly measurable biomarkers such as creatine kinase (CK) or lactate (Haller et al. 2023). High levels of CK and/or high‐sensitivity interleukin (IL)‐6 levels can result from inflammatory processes due to excessive exercise load (Romagnoli et al. 2016; Thorpe and Sunderland 2012). These biomarkers can be used to assess the acute internal load by assessing tissue‐ or organ‐specific fatigue, stress, damage, and/or recovery processes (Haller et al. 2023).

Effective monitoring and managing training load is essential not only for optimizing athletic performance but also for preventing overtraining and reducing the risk of injuries. Sports‐related injuries represent a significant healthcare burden and can lead to considerable psychological and motivational setbacks for athletes. Additionally, in professional sports, injuries have substantial economic repercussions (Ryan et al. 2019; Trentacosta 2020). The predominant etiology of sports‐related injuries is overuse injuries, resulting from repetitive stress and micro‐traumas, sometimes culminating in severe traumatic injuries (Tarnowski et al. 2022). Genetic information has been shown to be associated with performance‐related effects of sports training and predisposition to injuries (Guilherme et al. 2014; Varillas‐Delgado et al. 2022, 2023; Ginevičienė et al. 2022; Pfab et al. 2023). This study examines whether epigenetic markers, specifically DNA methylation levels, can be used to develop indicators related to injury risk.

Experimental evidence suggests that exercise acts as a significant stressor, driving various physiological adaptations in the body, including changes in epigenetic mechanisms. For example, research by Denham et al. demonstrated alterations in DNA methylation patterns in skeletal muscle following acute exercise, indicating a dynamic epigenetic response to exercise‐induced stress (Denham et al. 2014). A study by Rönn et al. revealed exercise‐induced changes in DNA methylation in adipose tissue, particularly in genes related to metabolism and inflammation (Rönn et al. 2013). Changes in methylation patterns can be interpreted biologically, for example, by using epigenetic clocks. Epigenetic clocks are DNAm‐based prediction methods for estimating age or mortality risk (Horvath 2013; Horvath et al. 2018; Lu et al. 2019). Second‐generation epigenetic clocks such as DNAm‐based GrimAge (DNAmGrimAge) and GrimAge2 (DNAmGrimAge2) predict future morbidity and mortality risk (Lu et al. 2019; Li et al. 2020). While DNAmGrimAge was trained on blood samples and an older population, DNAmGrimAge2 is also applicable to younger individuals and saliva samples (Lu et al. 2022). DNAmGrimAge is a composite biomarker (weighted linear combination) of seven DNAm surrogates of plasma proteins, a DNAm‐based estimator of smoking pack‐years, age, and sex. The seven DNAm‐based proteins comprise adrenomedullin (ADM), beta‐2‐microglobulin (B2M), cystatin C (Cystatin C), growth differentiation factor 15 GDF‐15, leptin (Leptin), plasminogen activator inhibitor 1 (PAI‐1), and tissue inhibitor metalloproteinases 1 (TIMP‐1). Version 2 of GrimAge leverages two additional DNAm‐based estimators of plasma proteins: CRP and hemoglobin A1C (logA1C) (Lu et al. 2022). Both versions of DNAmGrimAge perform well on predicting functional decline and onset of major age‐related conditions reliably across large diverse populations, including heart disease, cancer onset, multi‐modal measures of brain health, kidney disease, fatty liver, respiratory function, and more (Lu et al. 2019; Hillary et al. 2020, 2021; McCrory et al. 2021). Another epigenetic clock, which potentially has relevance to athlete performance, integrates fitness parameters into DNAmGrimAge2 to construct DNAm‐based FitAge (DNAmFitAge), a physical fitness age predictor. DNAmFitAge includes blood‐based DNAm biomarkers for fitness parameters like gait speed, maximum handgrip strength, forced expiratory volume in 1 s, and maximal oxygen uptake (McGreevy et al. 2023). Studies have revealed a correlation between physical fitness and biological age, as measured by DNA methylation age (DNAmFitAge) (Jokai et al. 2023).

Physically fit individuals tend to have a younger DNAmFitAge, which is associated with improved age‐related health outcomes. These individuals have a lower risk of mortality, coronary heart disease, and experience increased periods of disease‐free status, indicating better overall health maintenance as they age (McGreevy et al. 2023).

To the best of our knowledge, no studies have been conducted to investigate the short‐term dynamics of epigenetic clocks in professional soccer players. Furthermore, no data is available to evaluate possible relationships between epigenetic stress markers and injury events. In this exploratory study, we aimed to investigate the potential of newly developed epigenetic clocks such as DNAmGrimAge2 and DNAmFitAge as biomarkers to monitor and help manage athlete performance and to prevent unwanted side effects, like musculoskeletal damage or injuries.

Here we generated DNA methylation levels from saliva samples collected from professional soccer team members during a season, including time points with high and low physical stress. Widely used epigenetic clocks were compared to established injury/inflammation markers like CK, IL‐6, and C‐reactive protein (CRP) and tested whether DNAm‐based predictions thereof can be associated with injury occurrence.

## 2 Results

### 2.1 Vigorous Physical Activity Leads to a Rejuvenating Effect on Epigenetic Age Predictions

Saliva samples (*n* = 201) were collected from 24 members of a first‐league at nine different time points over a period of 6 months during the professional soccer seasons of 2021/22 and 2022/23 (Table 1).

**TABLE 1:** Overview of study participants. Age refers to the actual chronological age of participants at the beginning of the season.

	*N*		Mean age ± SD (years)
	Samples	Subjects	
All	156	19	26.23 ± 4.41
Forwarder	30	5	26.75 ± 2.40
Midfielder	54	6	26.42 ± 5.89
Defender	45	5	24.49 ± 2.11
Goalkeeper	27	3	29.51 ± 5.79
Supporting staff	45	5	42.61 ± 3.80

**FIGURE 1:**
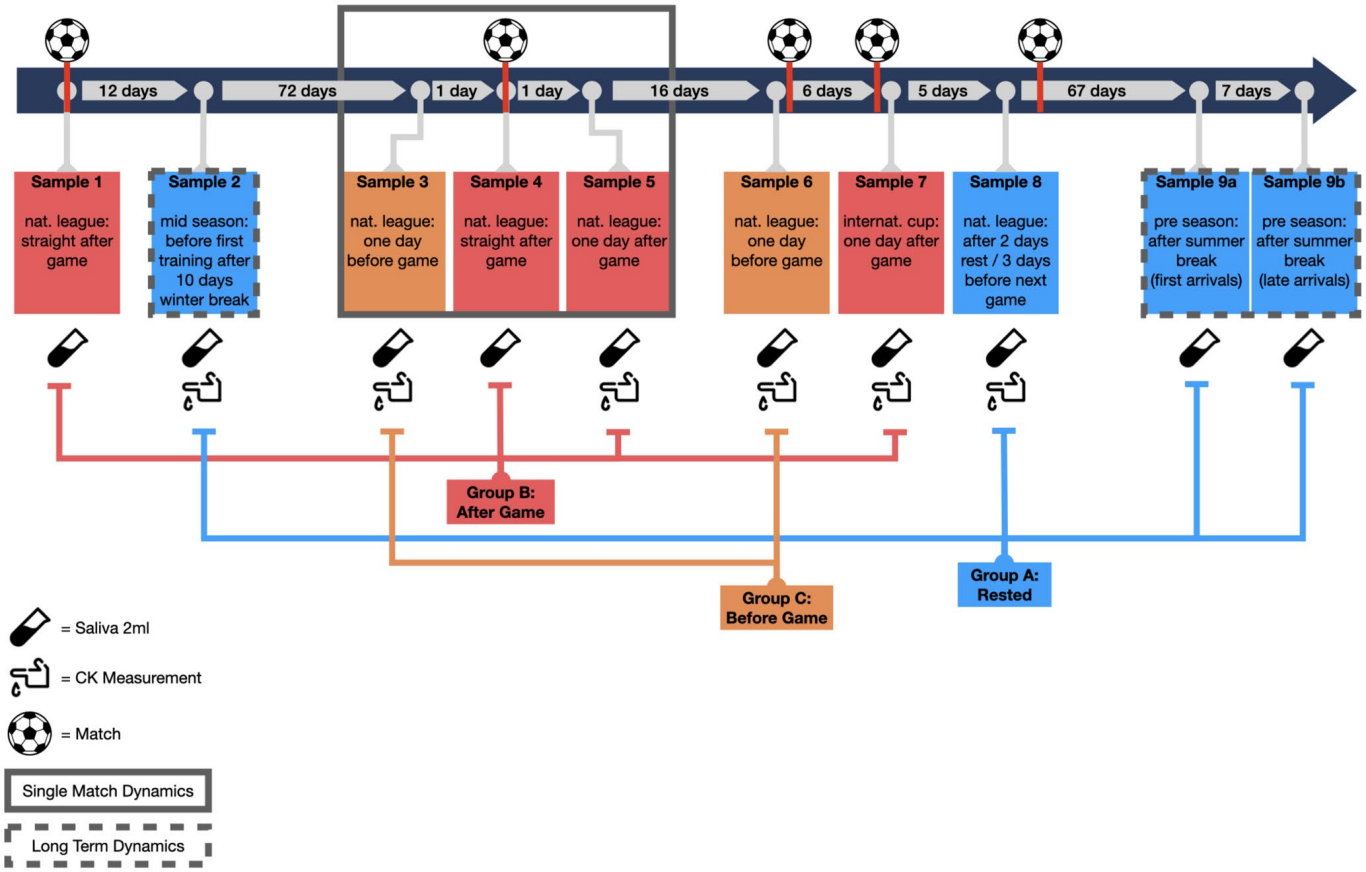
Sampling scheme and group assignment. The timeline indicates the individual measurement timepoints with respect to events and their intervals to each other. Events that involved strenuous physical exertion were denoted with a ball icon. Two different types of samples were collected from athletes as well as from supporting staff members serving as control group: saliva (indicated by saliva collection tube symbol) and blood samples for CK measurement (indicated by hand with blood drop symbol). DNA methylation data generated from saliva samples as well as CK measurement data was assigned to three groups: (A) rested, (B) straight or 1 day after match and (C) one day before match. Sample 9 was split into a and b, as it was taken after the summer break and the players involved returned from the break at two different times. In addition to examining short‐term effects after an intense game event (sample 3, 4 and 5; highlighted with a gray rectangle as “Single Match Dynamics”), data from sample 9 (end of season) were compared to data from sample 2 (start of season) to assess changes over a longer time frame (depicted with a dashed box as “Long Term Dynamics”).

Time points for sample collection were chosen to cover different physical stress states of athletes, so that samples can be grouped according to medium (before match) and high activity (immediately after game) phases. The state “rested” represents the training & recovery phase after match days (Figure 1). Using the HumanMethylationEPIC v1.0 BeadChip (Illumina, San Diego, CA) over 800,000 CpG sites within genomic DNA isolated from saliva were analyzed for their methylation status (DNAm). Various recently developed epigenetic markers of aging, known as epigenetic clocks, were calculated from the obtained methylation data. These include DNAmGrimAge2, DNAmFitAge, DNAmAge, and the Skin & Blood Clock (Lu et al. 2022; McGreevy et al. 2023; Horvath 2013; Horvath et al. 2018). To investigate the influence of strenuous physical activity on epigenetic age, we compared age predictions derived from samples (*n* = 156) obtained from athletes (*n* = 19, mean age ± standard deviation [SD]: 26.23 ± 4.41) divided into three groups representing different physical stress states based on the timing of collection (Figure 1). A control group was established using samples (*n* = 45) from 5 supporting staff members (mean age ± SD: 42.61 ± 3.80). The higher mean age for supporting staff should be noted and considered for comparison of baseline epigenetic age marker levels. Compared to active players, the supporting staff experienced the same environmental factors such as traveling, logistical challenges, and emotional distress, but lower levels of acute physical stress. GrimAge2 and FitAge predictions were calibrated (cal.) to the actual age range of players, referred to as GrimAge2 cal. and FitAge cal., respectively.

**FIGURE 2:**
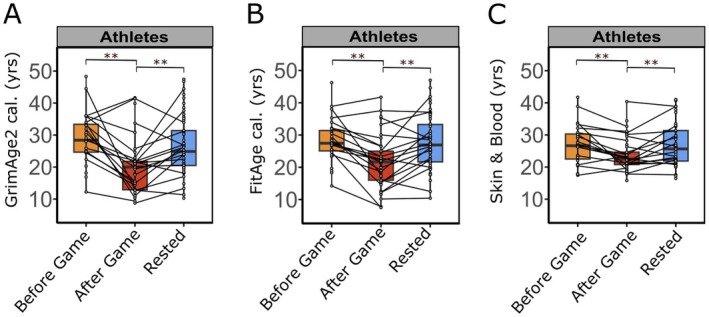
Intense physical activity causes rapid changes in biological age predictors. (A–C) Epigenetic profiles (DNAm) of saliva samples collected from athletes (*n* ≤ 19) during medium to low physical stress states (yellow: Before game, blue: Rested) or immediately after intensive physical activity (red: after game) were used to estimate biological/chronological age in years; The reference time point for all comparisons was After Game; (A) GrimAge2 cal. (before vs. after game *β* = −7.07, 95% CI: [−10.32, −3.71], *p* = 6.13e‐05, *f* ^2^ = 0.76; after game vs. rested *β* = 4.58, 95% CI: [1.62, 7.47], *p* = 0.00281, *f* ^2^ = 1.50); (B) FitAge cal. (before vs. after game *β* = −4.76, 95% CI: [−7.10, −2.36], *p* = 0.00016, *f* ^2^ = 1.50; after game vs. rested *β* = 3.88, 95% CI: [1.76, 5.96], *p* = 0.000476, f^2^ = 1.90); (C) Chronological age predictor Skin & Blood Clock (before vs. after game *β* = −2.99, 95% CI: [−4.53, −1.41], *p* = 0.000307, *f* ^2^ = 1.90; after game vs. rested *β* = 3.88, 95% CI: [2.48, 5.25], *p* = 3.19e‐07, *f* ^2^ = 2.30); (A–C) Each dot represents predicted age in years for a specific time point and player; Significance levels are indicated by ** (*p* ≤ 0.01); Statistical significance in predicted age differences was evaluated using a linear mixed‐effects model with chronological age and timepoints (before, after game, or rested) as fixed effects and player ID as well as batch number as a random effects; plots show median (bold line) with interquartile range (box) and 1.5‐fold interquartile range (whiskers); Cal.: GrimAge2 and FitAge predictions were calibrated to the actual age range of players.

Players' DNAm patterns showed significant changes when comparing samples collected before and after the game throughout the season (Figure 2A–C). These changes led to a considerable decrease in biological age predictors: DNAmGrimAge2 cal. decreased by 32% (*β* = −7.07, 95% CI: [−10.32, −3.71], *p* = 6.13e‐05), and DNAmFitAge cal. decreased by 18% (*β* = −4.76, 95% CI: [−7.10, −2.36], *p* = 1.6e‐04), and the Skin & Blood Clock decreased by 25.4% (*β* = −2.99, 95% CI: [−4.53, −1.41], *p* = 0.001).

Conversely, the biological ages of the supporting staff who were not exposed to the same intensity of physical stress remained constant before and after the game (Figures S1 and S2). As the sample size in the support staff group was limited (*n* = 5) it might be insufficient to detect meaningful effects. Both biological age predictors, DNAmGrimAge2 and DNAmFitAge, showed a transient change in athletes during competition, as their biological age returned to comparable values after the rest phase.

A more detailed investigation of player subgroups based on their assigned positions during games revealed that midfielders experienced the most significant rejuvenation effect (Figure S2A,C). The median DNAmGrimAge2 cal. of midfielders decreased by approximately 17.8 years (*β* = −10.00, 95% CI [−15.54, −4.46], *p* = 0.004) from before to after the game. This change was more pronounced compared to the moderate reductions observed in athletes playing forward (−11.3 years, *β* = −10.67, 95% CI [−16.76, −3.89], *p* = 0.008) and defenders (−5.3 years, *β* = −6.65, 95% CI [−12.54, −0.94], *p* = 0.074) (Figure S2A). Among the supporting staff members, physicians and physiotherapists exhibited a similar trend to athletes in terms of biological age, despite no significant overall change. Again, due to limited sample size in the support staff group, these results must be interpreted with caution.

### 2.2 Exercise‐Induced Epigenetic Changes Reflect Immunological Events

**FIGURE 3:**
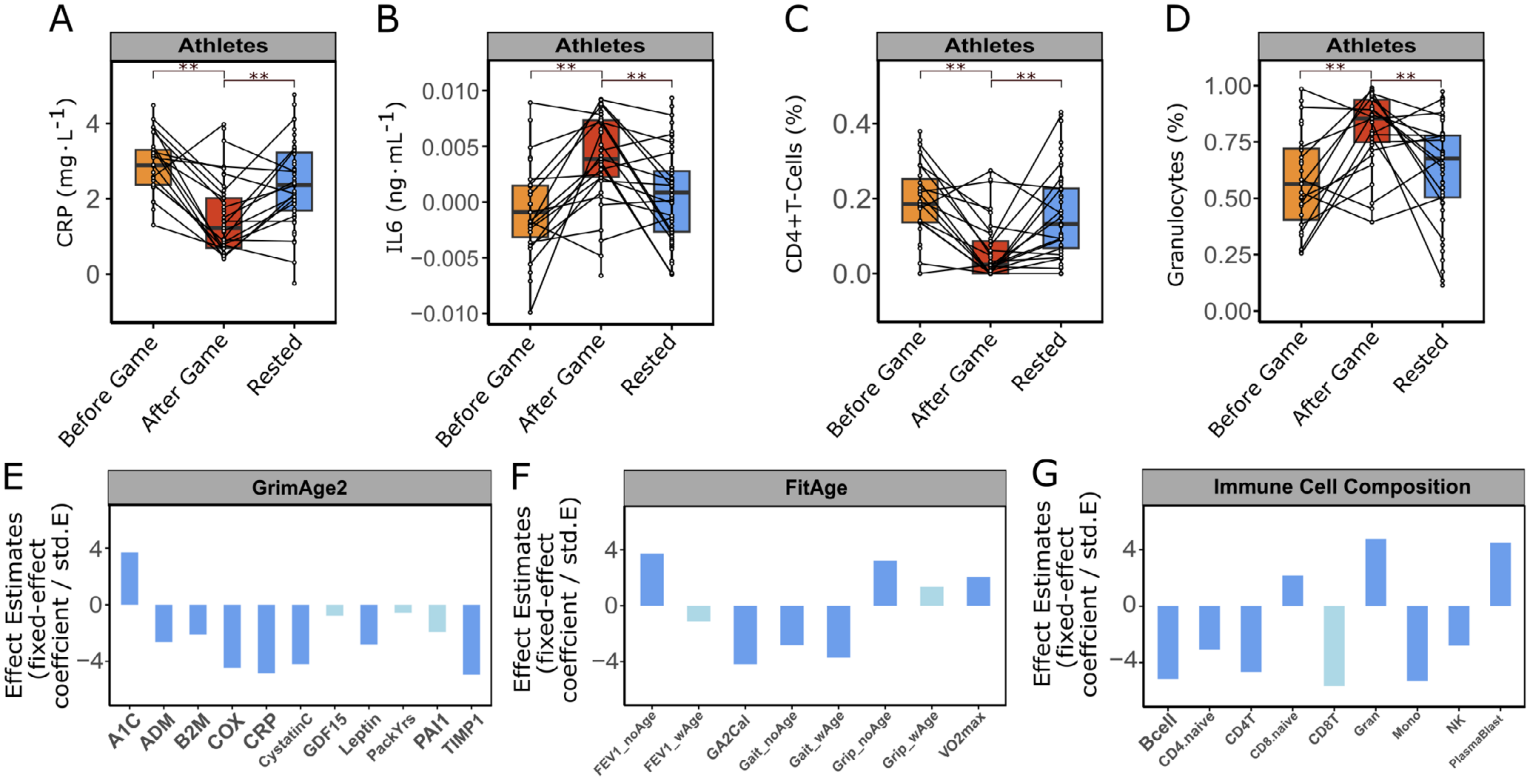
Activity‐induced changes in epigenetic age predictions reflect immunological events. (A–D) Boxplots illustrate changes of DNAm‐derived surrogate estimates for blood protein levels and relative immune cell abundance before and after physical activity (before/after game) and after recovery (rested) for inflammation markers from (*n* ≤ 19) athletes; The reference time point for all comparisons was After Game; (A) CRP (before vs. after game *β* = −1.24, 95% CI: [−1.74, −0.74], *p* = 4.81e‐06, *f* ^2^ = 0.38; after game vs. rested *β* = 0.74, 95% CI: [0.29, 1.18], *p* = 0.00162, *f* ^2^ = 0.27); (B) IL‐6 (before vs. after game *β* = 4.45e‐03, 95% CI: [0.0028, 0.0061], *p* = 6.78e‐07, *f* ^2^ = 0.28; after game vs. rested *β* = −2.71e‐03, 95% CI: [−0.0042, −0.0013], *p* = 0.00042, *f* ^2^ = 0.22); (C) active CD4 T‐Cells (before vs. after game *β* = −0.11, 95% CI: [−0.16, −0.06], *p* = 9.74e‐06, *f* ^2^ = 0.32; after game vs. rested *β* = 0.07, 95% CI: [0.03, 0.11], *p* = 0.00116, *f* ^2^ = 0.32); (D) Granulocytes (before vs. after game *β* = 0.23, 95% CI: [0.14, 0.33], *p* = 6.78e‐06, *f* ^2^ = 0.27; after game vs. rested *β* = −0.15, 95% CI: [−0.24, −0.07], *p* = 0.000648, *f* ^2^ = 0.27); (A–D) Each dot represents one sample from one participant; samples from the same participant are connected by lines across physical activity groups; Significant changes (*p*‐values) were tested using a linear mixed‐effects model with chronological age and timepoints (before, after game, or rested) as fixed effects and player ID as well as batch number as a random effects; (A–D) Significance levels are indicated by ** (*p* ≤ 0.01); (E–G) Importance analysis of various clock components including DNAm‐based plasma protein surrogate factor and blood immune cell composition estimates on determination of (E) DNAmGrimAge2, (F) DNAmFitAge and (G) DNAm based Immune Cell Composition in athletes after high intensity exercise (before vs. after game). Barplot shows standardized effect estimates of various clock components (fixed effects): Fixed‐effect coefficients (ß) divided by their respective standard errors (std.E) from a linear regression model fitting of the marker at before against after game timepoints. DNAm‐based predictor for blood levels of ADM: For adrenomedullin (ADM) (corr: −15.51, std.E: 5.84, *p* = 0.00924); B2M: DNAm of the beta‐2 microglobulin (B2M) gene (corr: −88973.44, std.E: 41988.41, *p* = 0.0365); CystatinC: DNAm of the cystatin C gene (corr: −54129.71, std.E: 12900.31, *p* = 5.79E‐05); GDF15: DNAm of the growth differentiation factor 15 (GDF15) gene (corr: −53.36, std.E: 68.41, *p* = 0.437); Leptin: DNAm of the leptin gene (corr: −4484.99, std.E: 1598.36, *p* = 0.006); PackYrs: DNAm‐based estimate of smoked cigarette packs per year (corr: −0.081, std.E: 0.14, *p* = 0.956); PAI1: DNAm of the plasminogen activator inhibitor‐1 gene (corr: −1464.13, std.E: 760.98, *p* = 0.0671); TIMP1: DNAm of the tissue inhibitor of metalloproteinases 1 gene (corr: −1238.74, std.E: 250.35, *p* = 2.96E‐06); COX: A composite clinical marker (corr: −1.07, std.E: 0.24, *p* = 2.13E‐05); A1C: DNAm‐based logarithmic transformation of glycated hemoglobin (HbA1c) (corr: 0.041, std.E: 0.011, *p* = 0.000345); CRP: DNAm‐based logarithmic transformation of C‐reactive protein (corr: −1.24, std.E: 0.26, *p* = 4.81E‐06); GA2Cal: Calibrated DNAm GrimAge, version 2, a predictor of biological age (corr: −7.07, std.E: 1.69, *p* = 6.13E‐05); VO2max: DNAm‐based estimate of maximal oxygen uptake (corr: 1.18, std.E: 0.57, *p* = 0.0413); Gait_noAge: DNAm‐based estimate of gait, excluding age as a factor (corr: −0.085, std.E: 0.030, *p* = 0.00593); Grip_noAge: DNAm‐based estimate of grip strength, excluding age as a factor (corr: 3.07, std.E: 0.95, *p* = 0.00167); FEV1_noAge: DNAm‐based estimate of forced expiratory volume in 1 s (FEV1), excluding age as a factor (corr: 0.35, std.E: 0.095, *p* = 0.000307); Gait_wAge: DNAm‐based estimate of gait, including age effects (corr: −0.087, std.E: 0.023, *p* = 0.000317); Grip_wAge: DNAm‐based estimate of grip strength, including age effects (corr: 0.41, std.E: 0.30, *p* = 0.18); FEV1_wAge: DNAm‐based estimate of FEV1, including age effects (corr: −0.051, std.E: 0.046, *p* = 0.268); Bcell: DNAm‐based proportion estimate of B cells (corr: −0.043, std.E: 0.0083, *p* = 1.24E‐06); CD4.naive: DNAm‐based estimate proportion of naive CD4^+^ T cells (corr: −94.36, std.E: 30.69, *p* = 0.00268); CD4T: DNAm‐based proportion of CD4^+^ T cells (corr: −0.11, std.E: 0.024, *p* = 9.74E‐06); CD8.naive: DNAm‐based proportion estimate of naive CD8^+^ T cells (corr: 25.43, std.E: 11.73, *p* = 0.0325); CD8T: DNAm‐based proportion estimate of CD8^+^ T cells (corr: –9.51E‐18, std.E: 1.68E‐18, *p* = 1); Gran: DNAm‐based proportion estimate of granulocytes (corr: 0.23, std.E: 0.049, *p* = 6.78E‐06); Mono: DNAm‐based proportion of monocytes (corr: −0.084, std.E: 0.016, *p* = 6.25E‐07); NK: DNAm‐based proportion estimate of natural killer cells (corr: −0.0070, std.E: 0.0025, *p* = 0.00613); PlasmaBlast: DNAm‐based proportion estimate of plasmablasts (corr: 0.50, std.E: 0.11, *p* = 1.98E‐05). Significance levels are indicated by dark blue (*p* ≤ 0.05) and light blue (*p* > 0.05). Cal.: GrimAge2 and FitAge predictions were calibrated to the actual age range of players.

Next, we aimed to investigate the epigenetic events underlying the substantial changes in DNAmGrimAge2 cal. following intense athletic workload. To this end, the influence of various plasma protein surrogate markers on changes in epigenetic age predictions induced by physical activity was examined (Figure 3). DNAmGrimAge2 consists of a group of nine DNAm‐based surrogates, which were trained to predict plasma protein levels. Among the six DNAmGrimAge2 covariates significantly associated with physical activity (*β* = −7.07, 95% CI: [−10.32, −3.71], *p* = 6.13e‐05), four correspond to proteins involved in inflammatory processes. Notably, a significant decrease (*β* = −1.24, 95% CI: [−1.74, −0.74], *p* = 4.81e‐06) in DNAm‐derived estimates of CRP levels, but an increase in IL‐6 (*β* = −4.45e‐03, 95% CI: [−0.0061, −0.0028], *p* = 6.78e‐07) was observed post‐competition among athletes, followed by a return to baseline levels after a period of rest (Figure 3A,B). In contrast, among supporting staff members, no significant alterations were observed in CRP or IL‐6 levels (Figure S1E). These findings suggest that activity‐induced modifications in epigenetic age predictions may mirror immunologic events associated with physical exertion. The elevation of inflammatory markers in athletes post‐competition could be attributed to the physiological stress and immune response triggered by high‐intensity exercise. This is also corroborated by changes at the cellular level. DNAm‐based estimation of immune cell composition in saliva samples indicates a significant decrease in CD4 T‐cells (−68%, *β* = −0.11, 95% CI: [−0.16, −0.06], *p* = 9.74e‐06), whereas granulocytes increased (+44%, *β* = 0.23, 95% CI: [0.14, 0.33], *p* = 6.78e‐06) comparing before to after game samples collected from athletes (Figure 3C,D). Notably, additional clocks that represent intrinsic cellular aging were also rejuvenated, including DNAmAge and IEAA, indicating that a reduction in epigenetic aging occurred beyond those attributable solely to changes in cell composition (Figure S3).

Our data suggest that epigenetic age predictions based on DNA methylation events can capture immunologic changes associated with physical activity and may have implications for comprehending the effects of strenuous exercise.

### 2.3 Dynamics of Short‐Term Effects of Physical Exertion on Epigenetic Age

**FIGURE 4:**
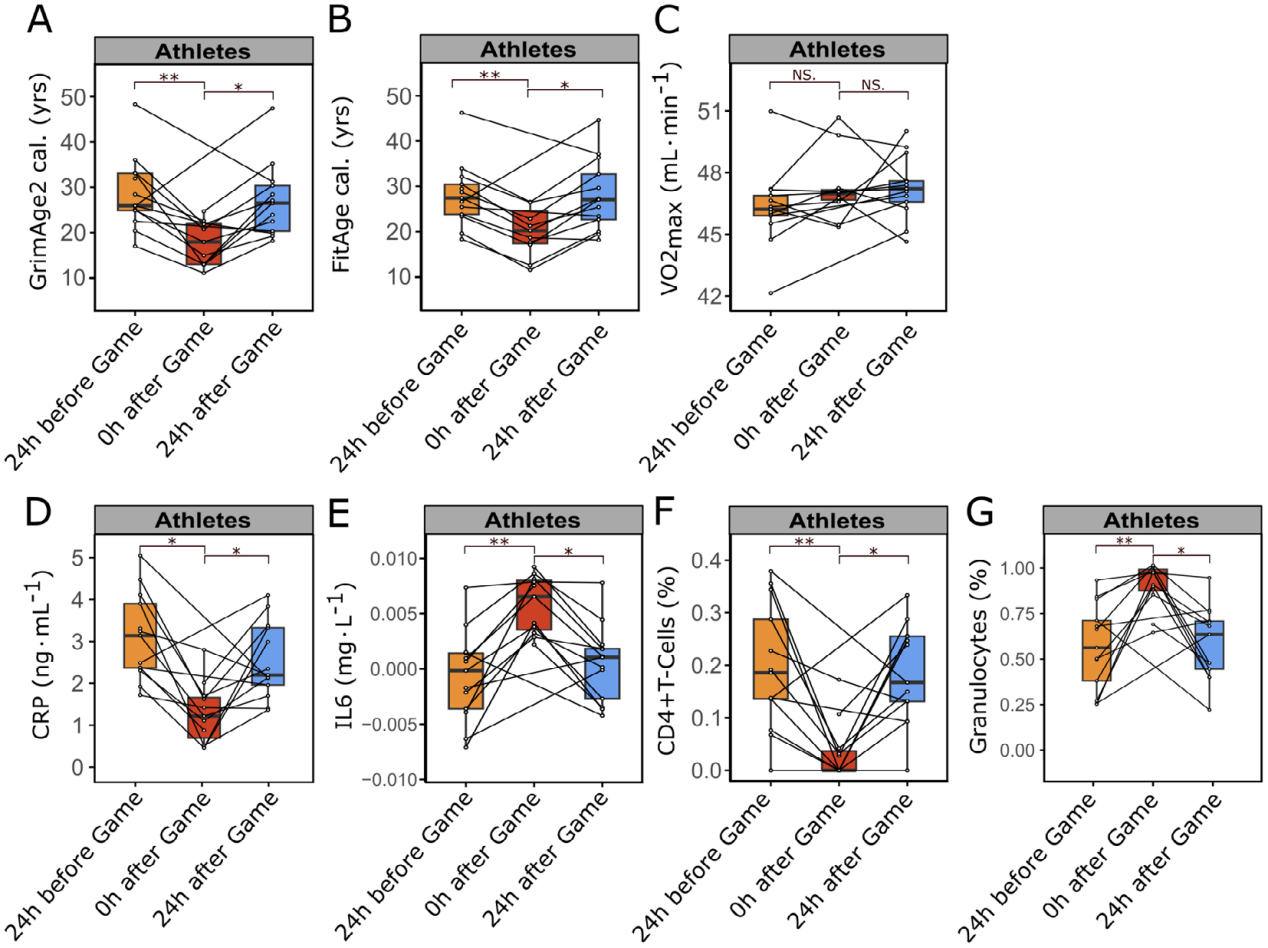
Instantaneous effects of high physical load on DNAm‐based age predictors and DNAm‐derived estimators of immunological factors. (A, B) Epigenetic profiles (DNAm) of saliva samples from *n* ≤ 19 athletes, collected during mid‐season game (samples 3, 4 and 5) 24 h before (24 h before game) or immediately after intensive physical activity (straight after game), were analyzed in addition to samples taken 24 h post high physical strain (24 h after game); The data were used to estimate DNAm‐based biological age (A) GrimAge2 cal. (24 h before vs. straight after game: *β* = −8.7379, 95% CI: [−13.6005, −3.7414], *p* = 0.0027, *f* ^2^ = 1.032; straight after vs. 24 h after game: *β* = 11.6929, 95% CI: [3.0752, 20.3649], *p* = 0.0166, *f* ^2^ = 1.032) and (B) FitAge cal. (24 h before vs. straight after game: *β* = −6.2199, 95% CI: [−9.5054, −2.8606], *p* = 0.00177, *f* ^2^ = 1.585; straight after vs. 24 h after game: *β* = 7.3302, 95% CI: [1.2466, 13.5292], *p* = 0.0327, *f* ^2^ = 1.585); Analysis of DNAm‐based endurance estimator (C) VO2max (24 h before vs. straight after game: *β* = 0.3935, 95% CI: [−0.6493, 1.4364], *p* = 0.480, *f* ^2^ = 0.480; straight after vs. 24 h after game: *β* = −1.1348, 95% CI: [−3.0412, 0.7912], *p* = 0.274, *f* ^2^ = 0.480), plasma protein surrogate factors (D) Methylation‐based estimator of CRP (24 h before vs. straight after game: *β* = −1.6024, 95% CI: [−2.3246, −0.8625], *p* = 0.00177, *f* ^2^ = 0.744; straight after vs. 24 h after game: *β* = 2.0116, 95% CI: [0.7267, 3.2883], *p* = 0.0327, *f* ^2^ = 0.744), and (E) Methylation‐based estimator of IL‐6 (24 h before vs. straight after game: *β* = 0.0063, 95% CI: [0.0042, 0.0084], *p* = 1.06e‐05, *f* ^2^ = 0.748; straight after vs. 24 h after game: *β* = −0.0056, 95% CI: [−0.0095, −0.0017], *p* = 0.0120, *f* ^2^ = 0.748), and immune cell type estimates for (F) CD4^+^ T‐Cells (24 h before vs. straight after game: *β* = −0.1507, 95% CI: [−0.2234, −0.0772], *p* = 0.00071, *f* ^2^ = 0.577; straight after vs. 24 h after game: *β* = 0.1794, 95% CI: [0.0467, 0.3103], *p* = 0.0160, *f* ^2^ = 0.577) and (G) Granulocytes (24 h before vs. straight after game: *β* = 0.3148, 95% CI: [0.1610, 0.4657], *p* = 0.00068, *f* ^2^ = 0.613; straight after vs. 24 h after game: *β* = −0.3899, 95% CI: [−0.6619, −0.1174], *p* = 0.0118, *f* ^2^ = 0.613); Each dot represents one sample from one participant, samples from the same participant are connected by line across physical activity groups, significant changes (*p*‐values) were tested using a linear mixed effect model with chronological age and timepoints (24 h before, straight after or 24 h after game) as fixed effects and player id as well as batch number as random effects. Significance levels are indicated by * (*p* ≤ 0.05), ** (*p* ≤ 0.01) and NS. (*p* > 0.05). Cal.: GrimAge2 and FitAge predictions were calibrated to the actual age range of players.

The reported effects of strenuous physical activity on DNA methylation‐based age predictors and immune‐related factors among athletes were derived from pooled samples collected throughout the season at varying intervals between high‐intensity matches, training sessions, and rest phases (Figures 1 and 2). For a detailed examination of the short‐term dynamics of epigenetic changes, we subsequently analyzed samples obtained during a 48‐h period encompassing a mid‐season game, which reflected a short sequence involving low (sample 3) to high load (sample 4) and a return to resting state (sample 5) (Figure 1). Understanding the temporal dynamic of effects can contribute to developing personalized strategies for optimizing athletic performance and mitigating potential health risks associated with intensive exercise. Our data demonstrate marked changes in epigenetic age predictors DNAmGrimAge2 cal. (−31%, *β* = −8.7379, 95% CI: [−13.6005, −3.7414], *p* = 0.003) and DNAmFitAge cal. (−18%, *β* = −6.2199, 95% CI: [−9.5054, −2.8606], *p* = 0.002) immediately following intensive physical activity (i.e., straight after the game) compared with 24 h before the game. This observation highlights the immediate impact of physical exertion on biological aging predictors (Figure 4A,B). Methylation‐based estimators of plasma proteins also showed significant variation in inflammatory responses after strenuous exercise (DNAmCRP: −50%, *β* = −1.6024, 95% CI: [−2.3246, −0.8625], *p* = 0.002 and DNAmIL‐6: +684%, *β* = 0.0063, 95% CI: [0.0042, 0.0084], *p* = 1.06e‐05) within 24 h (Figure 4D,E). DNAm‐based immune cell type estimates exhibit comparable transient alterations in response to physical exercise (Figure 4F,G). The observed changes were of a temporary nature, with values returning to baseline levels 24 h after the match. These values were similar to those measured 24 h before the game.

The control group of supporting staff had no significant changes in DNAmGrimAge2 cal., DNAmFitAge cal., DNAm‐based immune cell type alterations, and plasma protein surrogate factors (Figures S4 and S5B–D). Furthermore, we conducted a comparative analysis of samples collected at the conclusion of the season against those obtained at the beginning of the season (Figure S6). Following a 12‐month period, athletes exhibited only modest changes to epigenetic clocks that did not reach significance, including DNAmGrimAge2 cal. (−8.8%, *β* = −3.24, 95% CI: [−8.75, 2.27], *p* = 0.219), DNAmFitAge cal. (−2.7%, *β* = −1.97, 95% CI: [−5.76, 1.82], *p* = 0.292) and CRP level (−16%, *β* = −0.42, 95% CI: [−1.34, 0.51], *p* = 0.338) (Figure S6A–C).

### 2.4 Co‐Occurrence of DNA Methylation Changes With Injury in Athletes

The analysis of closely consecutive load changes during a midseason game (samples 3–5, Figure 1) suggested an interplay between physical activity, epigenetic alterations, and immune reactions, implying a dynamic relationship among these factors. During the investigation of the changes in DNA methylation age predictors, specifically DNAmGrimAge2 cal., DNAmFitAge cal., and several others, it was noted that these alterations typically followed a trend where pre‐game (baseline) levels were reduced significantly immediately post‐game, followed by a recovery back to baseline levels within the rest phase. Nonetheless, when comparing DNAmGrimAge2 cal. in players before the game (24 h prior, sample 3) to their rested state (24 h post‐game, sample 5), a variable trend was observed for certain players (Figure S7). Most players exhibited a moderate decrease in DNAmGrimAge2 cal., while a few players showed the opposite trend. Based on the fact that changes in DNAmGrimAge2 cal. were shown to correlate with immunologic events (Figure 3), which can be induced by major but also microtraumas (Gebhard et al. 2000; Schild et al. 2016), we took episodes of injuries affecting players close in time to the midseason match (Table 2) into consideration to delineate a possible source of the observed variation.

**TABLE 2:** Players with episodes of acute injury. Player column shows anonymized participant IDs; type of injury column shows injuries sustained during professional soccer season 2021/22 and 2022/23 matches or training sessions; timepoint column shows timepoints of injury.

Player	Type of injury	Timepoint
2	Musculoskeletal trauma	Sample 1, 7, 8
27	Musculoskeletal trauma	Samples 1–3
31	Musculoskeletal trauma	Sample 4
33	Musculoskeletal trauma	Samples 6–7

**FIGURE 5:**
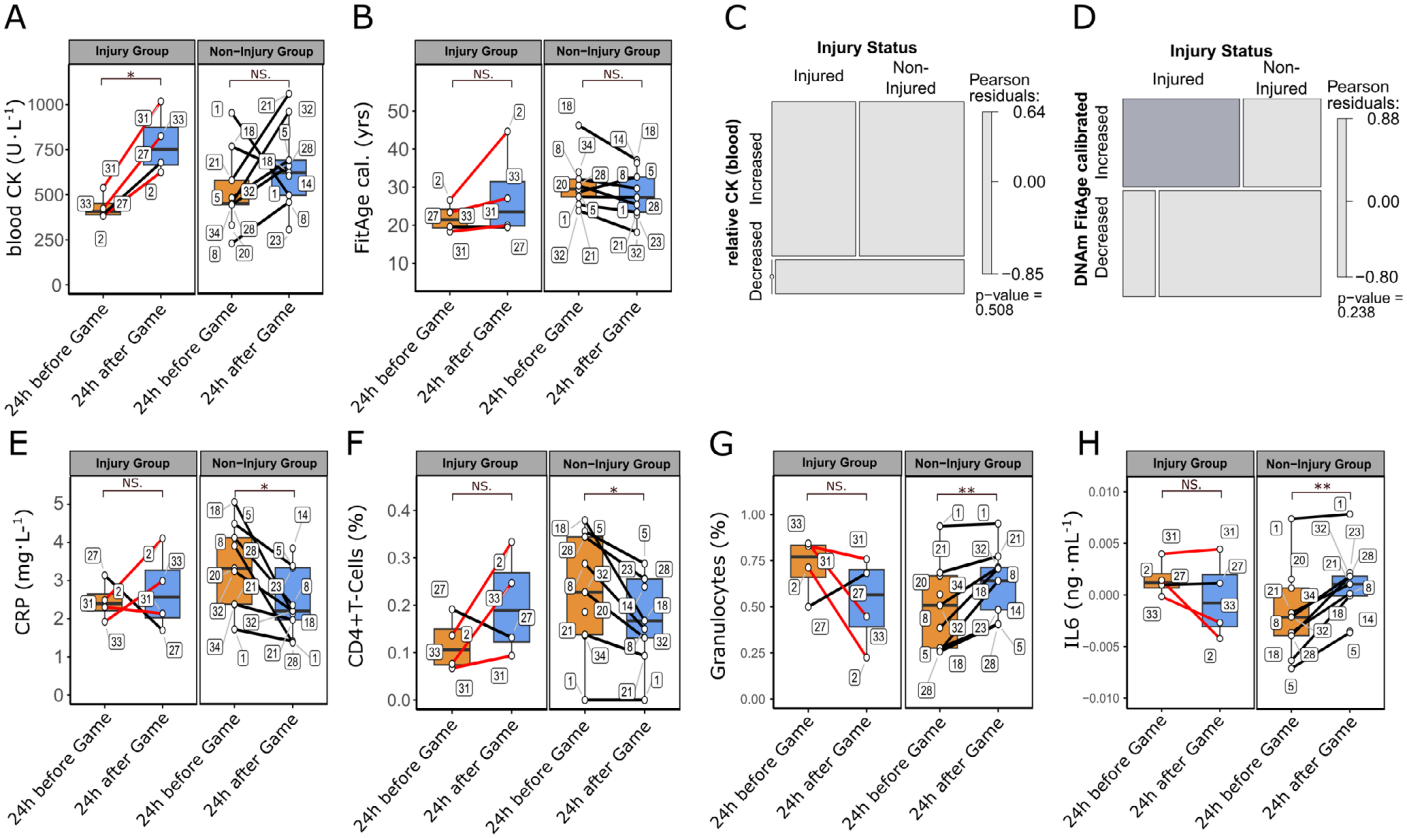
Differential patterns of biological age predictor FitAge in athletes are associated with events of injuries. In the course of a midseason match (sample 3 and 5) blood levels of inflammatory and tissue damage marker CK were determined and DNAm status of saliva samples assessed 24 h before and after the game. Individual players (*n* ≤ 19) indicated by number labels in plots were split into two groups depending on injury events (red lines: Players with history of injury during or after midseason match, black lines: Players without injury or injury before midseason match) with temporal proximity to the match day (see Table 2). Boxplots show (A) CK levels (Injury Group: 24 h before vs. 24 h after game *β* = 353.20, 95% CI: [260.44, 445.96], *p* = 0.013, *f* ^2^ = 8.435; Non‐Injury Group: 24 h before vs. 24 h after game *β* = 108.98, 95% CI: [−109.81, 327.66], *p* = 0.374, *f* ^2^ = 0.055), and (B) FitAge cal. in athletes (Injury Group: 24 h before vs. 24 h after game *β* = 5.79, 95% CI: [−2.72, 14.30], *p* = 0.257, *f* ^2^ = 0.776; Non‐Injury Group: 24 h before vs. 24 h after game *β* = −2.35, 95% CI: [−5.35, 1.24], *p* = 0.174, *f* ^2^ = 1.512). (C–D) Mosaic plots illustrate ratios of athletes in injury and non‐injury groups as well as the physical activity induced trend in (C) CK and (D) FitAge cal. Pearson residuals compare observed vs. expected number of players in subgroups and highlight significant increases (blue) and decreases (red) thereof. (E–G) saliva sample‐derived DNAm‐based estimators of (E) inflammatory marker CRP (Injury Group: 24 h before vs. 24 h after game *β* = 0.27, 95% CI: [−0.73, 1.27], *p* = 0.657, f^2^ = 0.230; Non‐Injury Group: 24 h before vs. 24 h after game *β* = −1.20, 95% CI: [−1.93, −0.15], *p* = 0.020, f^2^ = 0.315), (F) CD4^+^ T‐cells (Injury Group: 24 h before vs. 24 h after game *β* = 0.08, 95% CI: [−0.00, 0.17], *p* = 0.142, f^2^ = 1.147; Non‐Injury Group: 24 h before vs. 24 h after game *β* = −0.09, 95% CI: [−0.14, −0.03], *p* = 0.013, *f* ^2^ = 0.138), (G) Granulocytes (Injury Group: 24 h before vs. 24 h after game *β* = −0.19, 95% CI: [−0.41, 0.02], *p* = 0.175, *f* ^2^ = 0.845; Non‐Injury Group: 24 h before vs. 24 h after game *β* = 0.17, 95% CI: [0.06, 0.26], *p* = 0.010, *f* ^2^ = 0.154), and (H) IL‐6 (Injury Group: 24 h before vs. 24 h after game *β* = −0.02, 95% CI: [−0.07, 0.03], *p* = 0.222, f^2^ = 0.128; Non‐Injury Group: 24 h before vs. 24 h after game *β* = 0.08, 95% CI: [0.02, 0.15], *p* = 0.00194, *f* ^2^ = 0.192). Each dot represents one sample from one participant, samples from the same participant are connected by line across physical activity groups, significant changes (*p*‐values) were tested using a linear mixed effect model with chronological age and timepoints (24 h before or 24 h after game) as fixed and player ID as well as batch number as random effects and injury status instead of timepoints for intergroup comparison. Significance levels are indicated by * (*p* ≤ 0.05), ** (*p* ≤ 0.01) and NS. (*p* > 0.05). Cal.: GrimAge2 and FitAge predictions were calibrated to the actual age range of players.

To this end, blood samples collected during a midseason match (Figure 1, samples 3–5) were analyzed. In specific levels of CK, a marker of inflammation and tissue damage (Romagnoli et al. 2016), were measured. Additionally, DNA methylation status of saliva samples was assessed 24 h before and after the game to evaluate changes induced by physical activity. Based on their injury history (Table 2), players were divided into two groups: those affected by musculoskeletal trauma injuries and those without. In the injury group, CK levels significantly increased (*β* = 353.20, 95% CI: [260.44, 445.96], *p* = 0.013) on average 1.9‐fold in all athletes (Figure 5A). In contrast, the non‐injury group exhibited no discernible pattern in CK changes (*β* = 108.98, 95% CI: [−109.81, 327.66], *p* = 0.374, Figure 5A). Biological age predictor DNAmFitAge cal. showed a non‐significant trend of overall increase in the injury group (*β* = 5.79, 95% CI: [−2.72, 14.30], *p* = 0.257) and a decrease in the non‐injury group (*β* = −2.35, 95% CI: [−5.35, 1.24], *p* = 0.174) (Figure 5B). The only player (ID: 27) with a history of injury who did not show an increase in DNAmFitAge (Figure 5B, injury group panel) had suffered his injury at the beginning of the season (samples 1–3) and had already fully recovered at the time point of interest (midseason match, samples 3–5, Table 2). Comparing ratios of athletes with increasing or decreasing CK values and increasing or decreasing DNAmFitAge cal. within injury and non‐injury groups 24 h before and after the game, we found no statistically significant difference (Figure 5C,D, Table S1). Interestingly, although the injury group included only 4 injured players, and only 3 players injured at the time point of analysis, we saw a non‐significant increase of DNAmFitAge cal. comparing athlete ratios within the respective groups (*p* = 0.238) (Figure 5D). Excluding player 27 with an injury event earlier in the season (samples 1–3), who fully recovered at the time point of interest (midseason match, samples 3–5, Table 2) decreased the *p*‐value down to *p* = 0.071, making it borderline significant (data not shown). This obvious trend could not be seen looking at athlete ratios with increasing or decreasing CK values in the injury and non‐injury group (*p* = 0.508) (Figure 5C, Table S1). The small sample size of injured players (*n* = 4) limits the ability to draw any strong conclusions.

DNAm‐based plasma protein surrogate factors (Figure 5E,H) as well as immune cell composition type estimates (Figure 5F,G) resemble injury‐related trends in DNAmFitAge cal. and DNAmGrimAge2 cal. patterns. This demonstrates the ability of epigenetic clocks as ensemble biomarkers to integrate the various single DNAm predictors in reflecting immunologic events related to injury.

In summary, the results highlight the potential of using DNAm‐based biological age and inflammatory markers in predicting injury risk in athletes when exposed to high‐intensity physical load, which could lead to the development of fatigue monitoring and personalized injury prevention strategies.

## 3 Discussion

Our exploratory study aimed to investigate the influence of physical and environmental stressors on athletes’ epigenetic aging by assessing DNA methylation of saliva samples collected from professional soccer players throughout a season. Instead of analyzing individual markers, we used the methy‐lome to compute epigenetic clocks ‐ composite measures that predict age, health status, and other factors. Indeed, our study shows that both DNAmGrimAge2 and DNAmFitAge were able to capture transient short‐term changes in athletes within 48 h of a midseason competition as well as long‐term effects throughout the entire season.

Epigenetic regulation is involved in the physiological response to exercise training and might influence the predisposition to injury or disease (Tarnowski et al. 2022). Genes known to be highly involved in sports performance and exercise physiology include myocyte enhancer factor 2 (Potthoff et al. 2007) and slow‐twitching type I myosin heavy chain (Pandorf et al. 2009; Barrès et al. 2012), which are often the subject of exercise physiology research. DNA methylation is known to regulate gene expression and provides key links between genotype, phenotypic plasticity, and environment. In the context of athletic performance, a broad spectrum of environmental factors—the physical activity itself, nutrition, emotional challenges, and pre‐existing epigenetic signatures—can determine how an individual reacts to certain stressors (Ecker et al. 2018). Biological age, as measured by DNA methylation (DNAm)‐based epigenetic clocks, provides insights into individual aging trajectories beyond chronological age. However, these biomarkers are sensitive to a range of confounding factors, including genetic predisposition, physical fitness, diet, sleep, and inflammation, which modulate epigenetic aging in complex and tissue‐specific ways (Borrego‐Ruiz and Borrego 2024; Kusters and Horvath 2025). Genetics accounts for approximately 20%–25% of lifespan variability, while up to 80% stems from environmental and lifestyle factors (Plagg and Zerbe 2021). Physical activity induces beneficial hypomethylation of genes linked to mitochondrial function, metabolism, and muscle adaptation, particularly in skeletal muscle (*PGC1A*, *PDK4*, *NR4A3*) (García‐Giménez et al. 2024; Zheng et al. 2025). Dietary patterns and caloric restriction have been associated with delayed epigenetic aging, while poor nutrition and obesity (increased BMI) accelerate it (Levine et al. 2018; Kusters and Horvath 2025). Sleep deprivation and chronic psychosocial stress further promote epigenetic age acceleration, potentially via immune dysregulation and elevated pro‐inflammatory cytokines (Zannas et al. 2015; Ciaglia et al. 2025). Chronic inflammation is a key mediator of age‐related epigenetic changes. Epigenome‐wide association studies (EWAS) show strong associations between inflammatory biomarkers (CRP, IL‐6, TNF‐α) and methylation at aging‐related CpG sites (Ciaglia et al. 2025; Levine et al. 2018).

Physical exercise is increasingly recognized as a modulator of biological aging through its impact on DNA methylation, the foundational mechanism underlying epigenetic clocks such as DNAmGrimAge, DNAmPhenoAge, and the recently developed DNAmFitAge. In athletic and physically active populations, these clocks provide valuable insight into the physiological impact of training on aging trajectories. DNAmFitAge reflects cardiorespiratory and neuromuscular performance and reveals that individuals with high fitness levels demonstrate a biological age that is on average 1.5–2.0 years younger than their less fit counterparts (Jokai et al. 2023). These effects are supported by robust correlations with VO_2_max, BMI, HDL cholesterol, grip strength, and cognitive performance. Intervention studies further confirm that improvements in physical performance through structured exercise programs lead to measurable deceleration in biological aging, as indicated by reductions in DNAmGrimAge and DNAmPhenoAge (Loh et al. 2023; Hernandez Cordero et al. 2024; Seki et al. 2023). Genome‐wide analyses demonstrate that exercise induces extensive DNA methylation changes across tens of thousands of CpG sites in skeletal muscle and adipose tissue (Nitert et al. 2012; Rönn et al. 2013; Voisin et al. 2015; Seaborne et al. 2018), including hypomethylation of metabolic and angiogenic gene promoters such as PGC‐1α and MEF2A, indicating transcriptional activation of pathways related to endurance and hypertrophy. Notably, skeletal muscle appears to possess epigenetic memory, maintaining altered methylation patterns from prior training, which may enhance subsequent adaptations (Seaborne et al. 2018; Haupt et al. 2022). However, some studies have reported accelerated epigenetic aging in elite athletes, particularly power athletes, potentially due to chronic physiological stress (Spólnicka et al. 2018), though long‐term data from Olympic champions suggest overall decelerated epigenetic aging and reduced disease burden compared to nonathletes (Radák et al. 2025). The inconsistencies in observational studies may be attributed to differences in tissue specificity (e.g., muscle vs. blood), measurement techniques, and population heterogeneity (Galkin et al. 2023; Nagata et al. 2024). Mechanistically, exercise appears to exert anti‐inflammatory and immunomodulatory effects that influence DNA methylation of genes involved in oxidative stress, immune response, and cellular senescence (Fox et al. 2023; McGee and Hargreaves 2019). These processes are dynamic, with acute exercise eliciting more pronounced methylation shifts than chronic regimens, though residual epigenetic modifications persist and accumulate over time. Collectively, the evidence supports a model in which regular moderate‐to‐intense exercise induces favorable epigenetic remodeling across multiple tissues, decelerating biological aging and enhancing healthspan. The DNAmFitAge biomarker, in particular, holds promise as a tool to integrate fitness metrics into molecular aging models, though more longitudinal studies are needed to assess the persistence and generalizability of these effects across diverse populations. In line with the aforementioned observations, we could also show that moderate‐to‐intense physical activity in soccer players induces favorable epigenetic remodeling in saliva samples, decelerating short‐term and long‐term biological aging and possibly enhancing healthspan.

Physical exercise is known to induce adaptations in the immune system and metabolic changes, with upregulation of certain enzymatic and protein factors. If physical exercise is intense or extreme, the immune response is similar to that caused by other stressors, which might not be beneficial for the athlete. Blood levels of proteins, among other biomarkers that are part of the acute inflammatory response, including myoglobin, troponin, creatine kinase, lactate dehydrogenase, and C‐reactive protein, are considerably increased after endurance or highly demanding sport (Bernat‐Adell et al. 2021). DNAmGrimAge2 and DNAmFitAge consist of a group of nine DNAm‐based surrogates, which were trained to predict plasma protein levels. Among the six DNAmGrimAge2 covariates significantly associated with physical activity, four, including DNAmCRP, correspond to proteins involved in inflammatory processes. A multivariate linear regression analysis reported by Lu and colleagues revealed significant associations between saliva‐based AgeAccelGrim2 and clinically relevant measures, like high‐sensitivity C‐reactive protein, with lower values of DNAm logCRP and AccelGrim2 representing higher levels of physical functioning (Lu et al. 2022). CRP is an acute‐phase inflammatory marker primarily synthesized by hepatocytes under the influence of IL‐6 and plays a central role in the physiological response to muscle damage and systemic inflammation induced by exercise (Altarriba‐Bartes et al. 2020; Duarte et al. 2022). Moreover, its levels have been correlated with frailty, morbidity, and mortality (Allen and Golightly 2015), which is also predicted and correlated with GrimAge and GrimAge2. It was found that in trained athletes, when a single exercise protocol was applied, CRP temporarily increased as the acute‐phase response after exercise (Kasapis and Thompson 2005). Interestingly, our results showed an opposite overall trend when analysing short‐term effects of physical intermittent strenuous bouts, leading to immediate decreased DNAm‐based estimation of CRP levels, which normalize or even increase within a few hours after the game. In this context, although physical activity has been found to raise the CRP level acutely, it has been found that chronic physical activity (Kasapis and Thompson 2005). We could confirm the beneficial effect of long‐term physical activity by examining sample collection time points 2 and 9, representing time points before and after season, respectively. Following this 12‐month period, athletes exhibited a modest but insignificant decrease in the median DNAmGrimAge2, FitAge, and CRP level. Importantly, regular long‐term training may exert an anti‐inflammatory effect, with lower resting CRP levels observed in well‐conditioned athletes compared to sedentary individuals. This adaptation is associated with improvements in metabolic parameters, such as BMI, lipid profiles, and insulin sensitivity (Kostrzewa‐Nowak et al. 2015; Puzianowska‐Kuźnicka et al. 2016). However, protective adaptation appears load‐dependent; overuse or insufficient recovery may reverse this trend, exacerbating systemic inflammation. CRP typically rises 12–36 h post‐exercise as part of the acute‐phase inflammation. But still, there are many inconsistencies in the literature and contradictory findings depending on the type and timing of exercise. Bizheh and Jaafari showed CRP increased from 1.98 ± 0.46 mg/L at baseline to 2.67 ± 0.5 mg/L post‐exercise while there was no change within the control group (Bizheh and Jaafari 2011). In contrast, a meta‐analysis including 83 randomized and non‐randomized controlled trials indicated a decrease in CRP following exercise training. Furthermore, results suggested that engaging in exercise training is associated with a decrease in CRP levels regardless of the age or sex of the individual and that greater improvements in CRP levels occur with a decrease in body fat (Fedewa et al. 2017). Other studies reported no change in CRP immediately following and up to 24 h post‐exercise (Davis et al. 2008; Krüger et al. 1985; Mendham et al. 2011; Murtagh et al. 2005). These inconsistencies likely stem from measurement timing, as CRP and epigenetic clocks reflect responses to stressors and recovery processes that occur on different time scales. Methodological variability, different sample sources, and population‐based differences analyzing cohorts of distinct fitness levels, ages, and sex can be additional factors explaining some of the different outcomes of the studies. In the recent study, we predict CRP levels via methylation patterns, whereas other studies perform blood serum measurements. Additionally, genetic and epigenetic factors can influence CRP levels and responsiveness, with large‐scale EWAS identifying methylation signatures associated with chronic CRP elevations, especially in the context of obesity and smoking (Wielscher et al. 2022; Lundin et al. 2024). Our results show that both acute short‐ and intermittent long‐term physical activity can lower estimated CRP levels and might improve general fitness and biological age in soccer players.

Anaerobic training is typically used in a variety of sports settings and has been shown to have a significant impact on the composition of saliva (Ntovas et al. 2022). Because of the complexity of attributes required in sports such as soccer, they are considered randomized intermittent, dynamic, and skilled movement type sports. Research has consistently reported that acute bouts of endurance and resistance exercise can influence the migration of immune cells in the peripheral blood and saliva. Studies in saliva showed lymphocytosis immediately post exercise and a lymphopenia into the recovery period (Carlson et al. 2008; Carlson et al. 2017; Kraemer et al. 1996; Nieman and Pedersen 1999; Simonson and Jackson 2004). It seems that among both the young and elderly, an active lifestyle is generally linked to lower numbers and proportions of memory T cells and higher numbers and proportions of naïve T cells. This is partly supported by a recent systematic review, concluding that regular structured exercise increases the number of naïve T cells in peripheral blood at rest (Campbell and Turner 2018; Cao Dinh et al. 2017). Brown et al. characterized the T cell pool in young male and female adults classified as being very active well‐trained soccer players and compared to young adults classified as being untrained. Untrained individuals showed the highest proportions of CD4^+^ and CD8^+^ memory T cells and the lowest proportions of CD8^+^ naïve T cells, defined on the basis of CD57 and CD28 expression (Brown et al. 2014, 2015). In the present study, DNAm‐based estimation of immune cell composition in saliva samples indicates a significant increase in granulocytes, whereas CD4^+^ T‐cells decreased comparing before to after game samples collected from athletes (Figure 3C,D). These results could be confirmed and were even more pronounced looking at short‐term dynamics, comparing samples 3, 4, and 5. Long‐term effects (sample 2 vs. 9) showed the same tendencies with lower estimated CD4^+^ T‐cells. It has to be mentioned that increase and/or reduction in the frequency and function of lymphocytes and other immune cells in peripheral blood and saliva in the hours following vigorous and prolonged exercise does not necessarily reflect immune activation nor suppression. Instead, increasing numbers of granulocytes together with lymphopenia of CD4^+^ T‐cells and decreasing numbers of monocytes can represent a heightened state of immune surveillance and immune regulation driven by a preferential mobilization of cells to peripheral tissues. Granulocyte mobilization, particularly of neutrophils, is mediated by exercise‐induced elevations in IL‐6, granulocyte colony‐stimulating factor (G‐CSF), and hemodynamic stressors such as shear stress and catecholamine release (Jajtner et al. 2016; Markov et al. 2023). These cells, key effectors of the innate immune response, are recruited from bone marrow and vascular endothelium and subsequently trafficked to sites of muscle damage, where they contribute to the resolution of inflammation and tissue repair through phagocytosis, cytokine secretion, and crosstalk with other immune cells (Suzuki et al. 2002; Nieman et al. 1995). Furthermore, there is growing evidence from several studies in humans and rodents, indicating that exercise enhances, or at least does not suppress immune responses to *in vivo* challenge in younger and older individuals, supporting the contention that an acute bout of exercise has no detrimental immune consequences for health (Campbell and Turner 2018). This often misinterpreted decrease, often called immunosuppression, reflects a strategic redistribution of lymphocytes to peripheral tissues such as the respiratory mucosa, gastrointestinal tract, and damaged skeletal muscle, where enhanced immune surveillance is most needed (Dhabhar 2002; Brown et al. 2018). The post‐exercise decline in CD4^+^ T cells is modulated by stress hormones (e.g., cortisol, catecholamines), with evidence that subsets of regulatory T cells (Tregs) remain elevated post‐exercise, contributing to anti‐inflammatory regulation (Dorneles et al. 2019).

The physiological significance of this redistribution lies in the enhancement of host defense and tissue recovery. However, in periods of heavy training or competition without sufficient recovery, persistent lymphocytopenia and prolonged granulocyte elevation may reflect chronic inflammation, increased susceptibility to upper respiratory infections, and impaired performance (Morgado et al. 2020; Saidi et al. 2024). Long‐term, regular exercise contributes to the maintenance of immunological health by preserving naïve CD4^+^ T cell populations and limiting the accumulation of senescent memory T cells, which are associated with immunosenescence and systemic inflammation (Goldsmith et al. 2021). Higher cardiorespiratory fitness correlates with lower proportions of terminally differentiated CD4^+^ and CD8^+^ T cells, suggesting exercise limits immune aging (Spielmann et al. 2011; Hunt et al. 2023). All these findings point towards functional immunological adaptations that might support recovery and immune readiness upon transient increases in granulocytes and decreases in CD4^+^ T cells following exercise. These findings underscore the importance of monitoring immune markers in athletes, especially during congested training or competition periods, to optimize recovery, prevent illness, and sustain high performance.

Emerging evidence suggests that epigenetic mechanisms, including DNA methylation, histone modifications, and non‐coding RNAs, play a significant role in modulating injury risk, recovery, and tissue repair in athletes. These molecular processes regulate gene expression in response to physical stress, inflammation, and recovery demands, providing a biological framework for understanding individual variability in injury susceptibility (Tarnowski et al. 2022; Graham et al. 2021). Exercise‐induced changes in the methylome affect genes linked to muscle regeneration (*MyoD*, *PGC1α*), inflammation (*IL6*, *TNF*), and metabolic adaptation, with distinct signatures seen in resistance versus endurance training (Hunter et al. 2023). Athletes exposed to high workloads without adequate recovery may exhibit maladaptive epigenetic responses, contributing to chronic inflammation and increased soft tissue injury risk (Kusters and Horvath 2025; Potempa et al. 2025).

To illustrate the usefulness of epigenetic clocks not only in predicting biological and chronological aging, we analyzed the relationship between injury risk of individual athletes and changes in their DNAm‐based clock predictions within a season. For this purpose, we considered samples collected during a midseason game. With one exception, injury events occurred within the following 1–2 weeks of training and competition after the midseason game. The only player (ID: 27) with a history of injury who did not show an increase in DNAmGrimAge2 and DNAmFitAge had suffered his injury already at the beginning of the season (samples 1–3) and might have already fully recovered at the time point of measurement. Measurable parameters affected by exercise comprised changes in salivary cell numbers and cytokines and protein levels as an estimate using methylation data and protein levels in plasma. Our results show that the injury group followed an opposite trend compared to the overall trend of all samples analyzed within the season. Especially, CRP and cytokine levels reflect both acute and chronic inflammatory states and are also closely associated with exercise‐induced muscle damage and recovery (Souglis et al. 2015; Silva et al. 2022). As inflammation is tightly regulated by epigenetic mechanisms, especially in the early phases of tissue repair, disrupted regulation may impair recovery and predispose athletes to recurrent injuries. After the game, within the early resting phase, the injury group showed increasing CRP and decreasing IL‐6 levels estimated using methylation data. The non‐injury group showed decreasing CRP and increasing IL‐6 levels. These results point to a decreased fitness and health state within the early recovery phase of the injury group, whereas decreasing DNAmGrimAge2 and DNAmFitAge within the non‐injury group might indicate better and faster recovery of athletes after a game. In the context of professional soccer, CRP levels exhibit predictable responses to acute and chronic exercise loads. Notably, CRP concentrations commonly increase 24 h post‐match and typically return to baseline within 48 h, reflecting the resolution of acute inflammation. However, under congested match schedules—particularly with two games per week and ≤ 4 recovery days—CRP may remain elevated, suggesting cumulative inflammatory stress and incomplete tissue recovery (Altarriba‐Bartes et al. 2020; Duarte et al. 2022). The elevation of CRP in response to exercise is primarily driven by eccentric muscle contractions, common in soccer‐specific actions like sprinting, jumping, and directional changes. These actions lead to muscle microtrauma and initiate an inflammatory cascade, involving increased IL‐6, TNF‐α, and subsequent CRP synthesis. Peak CRP levels tend to correlate with the intensity and density of training and competition, with studies observing higher levels during pre‐season or after consecutive matches (Clemente et al. 2021; Souglis et al. 2015). Mechanistically, CRP acts as an opsonin, enhancing clearance of cellular debris, stimulating phagocytosis, and modulating both pro‐ and anti‐inflammatory responses, including complement activation and neutrophil recruitment (Potempa et al. 2025). Several proteins are affected in response to inflammatory processes, the majority showing increased levels shortly after an inflammatory reaction (Fedewa et al. 2017). IL‐6 concentration increases more than other cytokines during exercise, which might indicate muscle damage (Allen and Golightly 2015; Lightfoot et al. 2015). IL‐6 plasma concentrations are reportedly affected by factors other than intensity, such as type and time of exercise (Gleeson et al. 2012; Baumert et al. 2016). IL‐6 itself triggers the synthesis of hepatic acute‐phase protein CRP in tissue damage (Velissaris et al. 2017). High‐sensitivity CRP (hs‐CRP) measurements further reveal that chronic low‐grade inflammation may impair performance development and contribute to respiratory infections, especially in overreached or immunocompromised states (Blume and Wolfarth 2019). While acute CRP elevations are adaptive, persistent high levels across a competitive season may signal maladaptation, increased risk of illness, overtraining, or injury, particularly in conjunction with elevated CK and other markers of muscle damage (Becker et al. 2020; Yparraguirre Salcedo et al. 2024).

CK, a protein involved in muscle metabolism, is frequently used in sports medicine as an indirect marker of muscle damage, and its concentration is generally considered a physical stress marker (Moghadam‐Kia et al. 2016; Mougios 2007; Marqués‐Jiménez et al. 2016). CK levels have a significant variation with sex and ethnicity and with exercise type: eccentric exercise causes more muscle damage than concentric contractions of the same vigor (Baumert et al. 2016; Moghadam‐Kia et al. 2016). Analyzing CK blood serum levels of athletes within the injury and non‐injury group of the early recovery phase, we see elevated CK levels in both groups. Although a consistent pattern of elevated CK levels was observed in the injured group, the same pattern of CK changes in the uninjured group suggests that CK was not sufficient to discriminate between injured and uninjured athletes. Our results show that DNAm‐based estimation of CRP levels stays elevated within the recovery phase in athletes who experienced injuries within days after the game measurement, which may indicate that methylation‐based CRP estimation could be better in predicting injury events than CK. We conclude that high increases or stable CRP levels in athletes' blood, especially during recovery time, may indicate a clinically significant inflammation process related to microdamage to muscle tissue.

The results of our study indicate that epigenetic changes analyzed by biological clock estimators like DNAmGrimAge2 and DNAmFitAge have the potential to be utilized in prediction tools for injury predisposition in elite‐level soccer players or other intermittent strenuous sports. In the current study, we collected saliva samples, which are easily accessible within an athlete's daily schedule and therefore well accepted. This is a major advantage compared to more invasive blood sampling, which poses higher logistical challenges and a higher burden on athletes. In contrast, saliva samples display the highest variability in DNAm ages differences between EPICv1 and EPICv2, which can be attributed to the heterogeneous cell types in saliva, including epithelial cells and leukocytes, potentially leading to greater variations in DNAm ages. Blood samples tend to have higher quality DNA with less variability of stability, purity, and quantity compared to saliva. Cell composition adjustment in saliva and development of cell‐type deconvolution algorithms specific to saliva and the EPICv2 array would be essential for saliva to be used in cohort studies (Tay et al. 2025). This could further lead to the development of a non‐invasive personalized and more robust fatigue‐monitoring tool and injury prevention strategy than is available today.

From a practical perspective, combining and integrating epigenetic and inflammatory biomarkers into monitoring systems could enable early identification of at‐risk athletes, support personalized training and recovery plans, and optimize injury prevention strategies. In‐season and regular biomarker surveillance is essential during congested fixtures to avoid cumulative inflammatory burden, especially in players at higher risk of non‐contact injuries or illness. Pre‐season tracking can also inform training load progression and prevent maladaptive spikes due to excessive intensity. Ultimately, a nuanced understanding of epigenetic changes and protein biomarker (e.g., CRP) kinetics and their interpretation within the broader inflammatory context can help practitioners strike a critical balance between stimulus and recovery, ensuring optimal performance while minimizing injury and illness risk in elite soccer athletes. Elevated CRP post‐match or training should prompt adjustments in recovery protocols, including reduced training volume, increased rest, or implementation of individualized recovery strategies such as cryotherapy, nutrition, and sleep optimization.

Despite numerous observational studies (changes in biomarkers in response to exercise), to the best of our knowledge, there are no large cohort studies of athletes in which exercise was controlled/adjusted by changes in biomarker levels or in which biomarkers reliably predicted an outcome such as injury. Therefore, such studies are warranted to confirm our and others' results. As a single biomarker for training management and injury prevention is rather unrealistic with regard to the complex pattern of physiological responses in different sport areas, the development of a panel that includes aspects of inflammation, muscle status, and injury risk might allow for a more comprehensive picture of athletes and promote personalized training management within professional sports.

Our study is based on a unique dataset. However, we acknowledge several important limitations. First, the sample size is limited. A larger cohort with additional time points would allow for a more refined analysis of temporal dynamics. In particular, our investigation into the relationship between injury risk and methylation profiles is severely underpowered. Although the results are not statistically significant, we present them as a prototype to illustrate the type of analysis that should be conducted in future studies with greater statistical power. Second, the analyzed cohort is a highly adapted and trained group of athletes, making it difficult to find appropriate control groups for comparison. Therefore, an appropriate control group should be exposed to similar physical loads comparable to that of the athletes. In addition, the sample size of the control group should be increased, and ages should be matched. Third, our analysis of inflammatory markers (IL‐6 and CRP) relies on DNA methylation–based proxies rather than validated serum‐based measurements. While the observed increase in DNAm‐estimated IL‐6 aligns well with expectations, the acute decrease in DNAm‐estimated CRP is unexpected. This may reflect a limitation of the DNAm‐based CRP estimator, which may not be sensitive to short‐term inflammatory fluctuations. That said, we wish to briefly highlight a curious finding: the DNAm‐based estimate of CRP has been shown to be a better predictor of all‐cause mortality than serum‐based CRP in epidemiological studies. Fourth, our analysis of blood cell composition is also based on DNA methylation derived estimates, which have inherent limitations. Flow cytometry would provide more accurate and direct measurements. Furthermore, these estimation methods were developed for blood methylation data and may not be fully applicable to saliva. Nevertheless, the observed changes (namely, a decrease in CD4^+^ T cells and an increase in granulocytes) are consistent with findings from prior literature. Fifth, our study relies on bulk/saliva measurements. As such, the results may be confounded by changes in cell composition or other confounders.

Further research should aim to validate specific epigenetic signatures as predictive tools in sports medicine and explore their potential as targets for therapeutic intervention. With proper analysis and use of independent datasets, it can enable rigorous predictors and mechanistic understanding of short‐term effects of exercise and immune system changes.

## 4 Methodology

10 measurements were taken over an 187‐day period, with intervals depending on the respective match days and events (Figure 1). These were two regular league games and one game in the international cups before the matchday, on matchday within 1 h after the match or 1 day after the matchday and combined into three groups. Group A (Rested): all data from the measurement times that were taken in a rested state. Group B (After Game): all data from the measurement time points corresponding to a loaded state, which took place immediately after the match or on the following day. Group C (Before Game): before a game, where there is a certain baseline load, but the players should be sufficiently rested for the match day.

Data was collected in different ways due to practicability. For After Game measurement times, CK data was measured by samples of capillary blood 1 day after matches, and saliva samples (2 mL) were taken after matches using a saliva tube (Genefix/Isohelix). In Rested, CK measurement data was collected, and saliva samples (2 mL) were taken both before training. Except samples 9a and 9b, which were taken after the summer break with only one saliva sampling (2 mL).

### 4.1 Study and Statistical Design

Controlled longitudinal study with 10 measurements, comparing players with control staff. We set epigenetic clocks as the primary endpoint, before and after each stressor and over the whole season. Primarily using the GrimAge (Lu et al. 2019) and Skin & Blood Age clocks (Horvath et al. 2018). As a secondary outcome, using available metadata, we assessed the correlation of the correlation of all other key outcomes with GrimAge, including CK levels, 48 h training load, and self‐reported sleep and stress measurements.

We used multiple epigenetic biomarkers to assess epigenetic age before and after each stressor, and the whole season, including GrimAge, its component risk factors, and DNA methylation‐based immune cell subset predictors. To adjust for the longitudinal nature of the data, we used a linear mixed model analysis where the random intercept term corresponds to trial participant and a linear model where the dependent variable (trial‐completion epigenetic age) is regressed on epigenetic age at baseline and treatment status. Bonferroni correction was used to adjust for multiple comparisons.

We employed a linear mixed‐effects model, utilizing Satterthwaite's degrees of freedom method (lmerTest v3.1.3 R package) (Kuznetsova et al. 2017) to estimate the *p*‐values for each coefficient. Individual ID was treated as a random effect. Response variables varied depending on the analysis, encompassing epigenetic age, components of DNAmGrimAge2, or cellular estimates. Age, sample collection group (rested, straight/1 day after match, and 1 day before match), and plate numbers served as batch effect estimators. Notably, DNAmGrimAge2 components and cellular estimates were scaled.

### 4.2 Sample Collection

Fifteen players and five staff members of a professional men's soccer team participated in the study, providing informed written consent approved by the team's board of directors. Genetic and epigenetic analysis involved collecting mouth saliva samples at up to 10 different time points. These time points included periods after games and during rest, such as directly after game three within a week and before vacation, after 1 week of Christmas vacation 2021, during an extensive preseason training period, directly before a Euroleague game, directly after a Euroleague away game, the morning after that Euroleague away game, directly after a Euroleague game following a 2‐week antioxidant regimen, and directly after the last league game in May 2022. For saliva sample collection, participants were instructed to refrain from eating, drinking, smoking, brushing their teeth, or chewing gum for 30 min prior to collection. The collection involved spitting into a collection funnel attached to a saliva collection tube until the required volume was reached, excluding bubbles. The tube was then tightly capped, and the saliva was mixed with a stabilization solution by shaking the tube several times before storing at room temperature. Exclusion criteria: playtime < 60 min for players. Inclusion criteria for players: played right from the start for at least 60 min without pause. Inclusion criteria for supporting staff: part of the supporting staff during the match.

### 4.3 DNA Methylation and Epigenetic Clocks

DNA methylation data was generated using the Infinium MethylationEPIC BeadChip arrays (Illumina, San Diego, CA), with processing of methylation arrays by Eurofins Genomics. This enables the quantitative interrogation of more than 850,000 CpG methylation loci per sample, covering all designable RefSeq genes, with CpG Island shores, non‐island CpGs, CpG islands outside of coding regions, and miRNA promoter regions represented. The DNA methylation array assays involved bisulfite conversion of extracted DNA using a Zymo EZ DNA methylation kit, followed by DNA amplification, labeling, and arraying using MethylationEPIC BeadChip array kits, and scanning of the completed BeadChip arrays for final DNA methylation readout. The technology and methods described enable ready calculation of the most informative epigenetic aging clocks developed in the Horvath lab, including GrimAge (Lu et al. 2019), PhenoAge (Levine et al. 2018), the original pan‐tissue clock (Horvath 2013), and newer measures such as the Skin & Blood Age clock (Horvath et al. 2018).

Raw methylation signal intensities were obtained using the function read.metharray.exp. from the minfi v1.40.0 R package, followed by linear dye bias correction and noob background correction to address technical variation in background fluorescence signal (Aryee et al. 2014). Specifically, the *β*‐value was computed from the intensity of methylated and unmethylated sites as the ratio of fluorescent signals. *β*‐values were utilized in all analyses. Subsequently, we computed several human epigenetic biomarkers of aging (epigenetic clocks) and estimated cell compositions based on blood methylation data: the pan‐tissue epigenetic age (referred to as DNAmAge) (Horvath 2013); Hannum's blood‐based DNAm age (DNAmHannum) (Horvath 2013); skin and blood clock (DNAmAgeSkinClock) (Horvath et al. 2018); DNAm of surrogate markers of telomere length (DNAmTL) (Lu et al. 2019); DNAmPhenoAge (Levine et al. 2018); the mortality risk estimator DNAmGrimAge and its components (Lu et al. 2019); DNAmGrimAge2 (Lu et al. 2022); DNAmFitAge (McGreevy et al. 2023). As DNAmFitAge and DNAmGrimAge2 were trained using blood samples, they tend to overestimate epigenetic age when applied to saliva samples. These two clocks were calibrated using a Clock Foundation trained model trained model on a reference database consisting of 1154 healthy untreated samples with known sex and age (age varies from 18 to 91) from different sample sources (blood, saliva, buccal). The trained model estimates the sample source, age, and sex of an individual to correct the overestimation trend of the original clocks. Data analysis conducted through the Clock Foundation enables many additional quality control measures to be performed, as well as the calculation of DNA methylation‐based surrogate measures, such as predicting the status of the immune system. Immune cell counts were inferred from DNA methylation using the Houseman estimation method (Houseman et al. 2012) and Horvath's blood cell count estimation approach, as described in Horvath and Levine (2015). One reason a major focus was on GrimAge2 is that it was tested extensively in saliva samples. The samples were also tested using saliva cell‐type deconvolution methods described by Middleton et al. (2022) and found to contain mostly leukocytes (averaging 97% predicted composition) rather than epithelial cells (< 10% predicted composition on average).

### 4.4 Additional Study Monitoring

In addition to the genetic and epigenetic analyses, a range of supplementary tests and assessments were conducted to enhance the comprehensive evaluation of the participants. Capillary blood testing was performed to measure Creatine Kinase (CK), which serves as a reliable measure of muscle strain, comparable to lactate measurements obtained from spiroergometry testing. CK levels were assessed daily to enable effective monitoring of muscle stress and provide valuable insights into the participants' training status. Daily recordings of creatine kinase levels were stored in SAP SportsOne or Excel for subsequent analysis.

## Author Contributions

F.P., R.T.B., and S.H. conceived and planned the study design. C.H., T.B., F.P., J.S., and W.B. conceived and planned the experiments. C.H., T.B., J.S., and F.P. carried out the sample collection. D.L. processed the samples. R.T.B., J.G., and M.M. developed the algorithms and performed the computations. T.K., R.Z., and M.N. verified the analytical calculations. T.K. and R.Z. contributed to the interpretation of the results. T.K. and R.Z. took the lead in writing the manuscript. All authors provided critical feedback and helped shape the research, analysis, and manuscript.

## Disclosure

Permission Statement: Robert T. Brooke, Thomas Kocher, Roland Zauner, Juozas Gordevicius, Milda Milčiūtė, Marc Nowakowski, Christian Haser, Thomas Blobel, Johanna Sieland, Daniel Langhoff, Winfried Banzer, Steve Horvath, and Florian Pfab have all approved the submission of this manuscript.

## Ethics Statement

The study was approved by the local Ethics Committee of the Faculty of Psychology and Sport Science, Goethe‐University Frankfurt/Main, Germany. The investigation was conducted according to the ethical standards set by the Declaration of Helsinki (World Medical Association Declaration of Helsinki: ethical principles for medical research involving human subjects. JAMA. 2013;310(20):2191–4. doi:10.1001/jama.2013.281053. Cited in: PubMed; PMID 24141714).

## Conflicts of Interest

The Regents of the University of California are the sole owners of patents and patent applications directed at epigenetic biomarkers for which Steve Horvath is a named inventor; Steve Horvath is a founder and paid consultant of the non‐profit Epigenetic Clock Development Foundation that licenses these patents. Steve Horvath is a Principal Investigator at Altos Labs, Cambridge Institute of Science, a biomedical company that works on rejuvenation. Florian Pfab is a shareholder of DNAthlete AG. Florian Pfab & Marc Nowakowski are employees of DNAthlete Austria GmbH.

## Supporting information


Data S1.

## Data Availability

To protect the confidentiality of the participants, we will not be able to distribute full data in the public domain. Anonymized summary statistics and epigenetic biomarker results will be made available to researchers upon request.

## References

[acel70182-bib-0001] Allen, K. D. , and Y. M. Golightly . 2015. “State of the Evidence.” Current Opinion in Rheumatology 27, no. 3: 276–283. 10.1097/BOR.0000000000000161. 25775186 PMC4405030

[acel70182-bib-0002] Altarriba‐Bartes, A. , J. Peña , J. Vicens‐Bordas , R. Milà‐Villaroel , and J. Calleja‐González . 2020. “Post‐Competition Recovery Strategies in Elite Male Soccer Players. Effects on Performance: A Systematic Review and Meta‐Analysis.” PLoS One 15, no. 10: e0240135. 10.1371/journal.pone.0240135. 33007044 PMC7531804

[acel70182-bib-0003] Aryee, M. J. , A. E. Jaffe , H. Corrada‐Bravo , et al. 2014. “Minfi: A Flexible and Comprehensive Bioconductor Package for the Analysis of Infinium DNA Methylation Microarrays.” Bioinformatics 30, no. 10: 1363–1369. 10.1093/bioinformatics/btu049. 24478339 PMC4016708

[acel70182-bib-0004] Barnes, C. , D. T. Archer , B. Hogg , M. Bush , and P. S. Bradley . 2014. “The Evolution of Physical and Technical Performance Parameters in the English Premier League.” International Journal of Sports Medicine 35, no. 13: 1095–1100. 10.1055/s-0034-1375695. 25009969

[acel70182-bib-0005] Barrès, R. , J. Yan , B. Egan , et al. 2012. “Acute Exercise Remodels Promoter Methylation in Human Skeletal Muscle.” Cell Metabolism 15, no. 3: 405–411. 10.1016/j.cmet.2012.01.001. 22405075

[acel70182-bib-0006] Baumert, P. , M. J. Lake , C. E. Stewart , B. Drust , and R. M. Erskine . 2016. “Genetic Variation and Exercise‐Induced Muscle Damage: Implications for Athletic Performance, Injury and Ageing.” European Journal of Applied Physiology 116, no. 9: 1595–1625. 10.1007/s00421-016-3411-1. 27294501 PMC4983298

[acel70182-bib-0007] Becker, M. , B. Sperlich , C. Zinner , and S. Achtzehn . 2020. “Intra‐Individual and Seasonal Variation of Selected Biomarkers for Internal Load Monitoring in U‐19 Soccer Players.” Frontiers in Physiology 11: 838. 10.3389/fphys.2020.00838. 32848822 PMC7417431

[acel70182-bib-0008] Bernat‐Adell, M. D. , E. J. Collado‐Boira , P. Moles‐Julio , et al. 2021. “Recovery of Inflammation, Cardiac, and Muscle Damage Biomarkers After Running a Marathon.” Journal of Strength and Conditioning Research 35, no. 3: 626–632. 10.1519/JSC.0000000000003167. 31045685

[acel70182-bib-0009] Bizheh, N. , and M. Jaafari . 2011. “The Effect of a Single Bout Circuit Resistance Exercise on Homocysteine, Hs‐CRP and Fibrinogen in Sedentary Middle Aged Men.” Iranian Journal of Basic Medical Sciences 14, no. 6: 568–573. 23493183 PMC3586852

[acel70182-bib-0010] Blume, K. , and B. Wolfarth . 2019. “Identification of Potential Performance‐Related Predictors in Young Competitive Athletes.” Frontiers in Physiology 10: 1394. 10.3389/fphys.2019.01394. 31803061 PMC6872676

[acel70182-bib-0011] Borrego‐Ruiz, A. , and J. J. Borrego . 2024. “Epigenetic Mechanisms in Aging: Extrinsic Factors and Gut Microbiome.” Genes 15, no. 12: 1599. 10.3390/genes15121599. 39766866 PMC11675900

[acel70182-bib-0125] Brown, F. F. , A. B. Bigley , J. C. Ross , E. C. LaVoy , R. J. Simpson , and S. D. Galloway . 2015. “T‐Lymphocyte Populations Following a Period of High Volume Training in Female Soccer Players.” Physiology & Behavior 152, no. Pt A: 175–181. 10.1016/j.physbeh.2015.09.027. Epub 2015 Sep 30. 26432452

[acel70182-bib-0124] Brown, F. F. , A. B. Bigley , C. Sherry , et al. 2014. “Training Status and Sex Influence on Senescent T‐Lymphocyte Redistribution in Response to Acute Maximal Exercise.” Brain, Behavior, and Immunity 39: 152–159. 10.1016/j.bbi.2013.10.031. Epub 2013 Nov 4. 24200513

[acel70182-bib-0012] Brown, F. F. , J. P. Campbell , A. J. Wadley , J. P. Fisher , S. Aldred , and J. E. Turner . 2018. “Acute Aerobic Exercise Induces a Preferential Mobilisation of Plasmacytoid Dendritic Cells Into the Peripheral Blood in Man.” Physiology & Behavior 194: 191–198. 10.1016/j.physbeh.2018.05.012. 29763678

[acel70182-bib-0122] Campbell, J. P. , and J. E. Turner . 2018. “Debunking the Myth of Exercise‐Induced Immune Suppression: Redefining the Impact of Exercise on Immunological Health Across the Lifespan.” Frontiers in Immunology 9: 648. 10.3389/fimmu.2018.00648. 29713319 PMC5911985

[acel70182-bib-0123] Cao Dinh, H. , I. Beyer , T. Mets , et al. 2017. “Effects of Physical Exercise on Markers of Cellular Immunosenescence: A Systematic Review.” Calcified Tissue International 100, no. 2: 193–215. 10.1007/s00223-016-0212-9. Epub 2016 Nov 19. 27866236

[acel70182-bib-0117] Carlson, L. A. , S. Headley , J. DeBruin , A. T. Tuckow , A. J. Koch , and R. W. Kenefick . 2008. “Carbohydrate Supplementation and Immune Responses After Acute Exhaustive Resistance Exercise.” International Journal of Sport Nutrition and Exercise Metabolism 18, no. 3: 247–259. 10.1123/ijsnem.18.3.247. 18562773

[acel70182-bib-0118] Carlson, L. A. , M. A. Lawrence , K. LeCavalier , and A. J. Koch . 2017. “Salivary Lymphocyte Responses Following Acute Anaerobic Exercise in a Cool Environment.” Journal of Strength and Conditioning Research 31, no. 5: 1236–1240. 10.1519/JSC.0000000000001593. 27537409

[acel70182-bib-0013] Ciaglia, E. , F. Montella , V. Lopardo , et al. 2025. “The Genetic and Epigenetic Arms of Human Ageing and Longevity.” Biology 14, no. 1: 92. 10.3390/biology14010092. 39857322 PMC11762130

[acel70182-bib-0014] Clemente, F. M. , F. T. González‐Fernández , H. I. Ceylan , et al. 2021. “Blood Biomarkers Variations Across the Pre‐Season and Interactions With Training Load: A Study in Professional Soccer Players.” Journal of Clinical Medicine 10, no. 23: 5576. 10.3390/jcm10235576. 34884288 PMC8658324

[acel70182-bib-0016] Davis, J. , M. Murphy , T. Trinick , E. Duly , A. Nevill , and G. Davison . 2008. “Acute Effects of Walking on Inflammatory and Cardiovascular Risk in Sedentary Post‐Menopausal Women.” Journal of Sports Sciences 26, no. 3: 303–309. 10.1080/02640410701552906. 17943596

[acel70182-bib-0018] Denham, J. , F. Z. Marques , B. J. O'Brien , and F. J. Charchar . 2014. “Exercise: Putting Action Into Our Epigenome.” Sports Medicine 44, no. 2: 189–209. 10.1007/s40279-013-0114-1. 24163284

[acel70182-bib-0127] Dhabhar, F. S. 2002. “Stress‐Induced Augmentation of Immune Function–The Role of Stress Hormones, Leukocyte Trafficking, and Cytokines.” Brain, Behavior, and Immunity 16, no. 6: 785–798. 10.1016/s0889-1591(02)00036-3. 12480507

[acel70182-bib-0020] Dorneles, G. P. , I. M. da Silva , A. Peres , and P. R. T. Romão . 2019. “Physical Fitness Modulates the Expression of CD39 and CD73 on CD4^+^ CD25^−^ and CD4^+^ CD25^+^ T Cells Following High Intensity Interval Exercise.” Journal of Cellular Biochemistry 120, no. 6: 10726–10736. 10.1002/jcb.28364. 30663116

[acel70182-bib-0021] Duarte, W. , J. L. Rodrigues Júnior , L. V. Paula , et al. 2022. “C‐Reactive Protein and Skin Temperature of the Lower Limbs of Brazilian Elite Soccer Players Like Load Markers Following Three Consecutive Games.” Journal of Thermal Biology 105: 103188. 10.1016/j.jtherbio.2022.103188. 35393043

[acel70182-bib-0022] Ecker, S. , V. Pancaldi , A. Valencia , S. Beck , and D. S. Paul . 2018. “Epigenetic and Transcriptional Variability Shape Phenotypic Plasticity.” BioEssays 40, no. 2: 1700148. 10.1002/bies.201700148. 29251357

[acel70182-bib-0023] Fedewa, M. V. , E. D. Hathaway , and C. L. Ward‐Ritacco . 2017. “Effect of Exercise Training on C Reactive Protein: A Systematic Review and Meta‐Analysis of Randomised and Non‐Randomised Controlled Trials.” British Journal of Sports Medicine 51, no. 8: 670–676. 10.1136/bjsports-2016-095999. 27445361

[acel70182-bib-0025] Fox, F. A. U. , D. Liu , M. M. B. Breteler , and N. A. Aziz . 2023. “Physical Activity Is Associated With Slower Epigenetic Ageing‐Findings From the Rhineland Study.” Aging Cell 22, no. 6: e13828. 10.1111/acel.13828. 37036021 PMC10265180

[acel70182-bib-0027] Galkin, F. , O. Kovalchuk , D. Koldasbayeva , A. Zhavoronkov , and E. Bischof . 2023. “Stress, Diet, Exercise: Common Environmental Factors and Their Impact on Epigenetic Age.” Ageing Research Reviews 88: 101956. 10.1016/j.arr.2023.101956. 37211319

[acel70182-bib-0028] García‐Giménez, J. L. , I. Cánovas‐Cervera , and F. V. Pallardó . 2024. “Oxidative Stress and Metabolism Meet Epigenetic Modulation in Physical Exercise.” Free Radical Biology and Medicine 213: 123–137. 10.1016/j.freeradbiomed.2024.01.008. 38199289

[acel70182-bib-0029] Gebhard, F. , H. Pfetsch , G. Steinbach , W. Strecker , L. Kinzl , and U. B. Brückner . 2000. “Is Interleukin 6 an Early Marker of Injury Severity Following Major Trauma in Humans?” Archives of Surgery 135, no. 3: 291–295. 10.1001/archsurg.135.3.291. 10722030

[acel70182-bib-0030] Ginevičienė, V. , A. Utkus , E. Pranckevičienė , E. A. Semenova , E. C. R. Hall , and I. I. Ahmetov . 2022. “Perspectives in Sports Genomics.” Biomedicine 10, no. 2: 298. 10.3390/biomedicines10020298. PMC8869752 35203507

[acel70182-bib-0031] Gleeson, M. , N. Bishop , M. Oliveira , T. McCauley , P. Tauler , and A. S. Muhamad . 2012. “Respiratory Infection Risk in Athletes: Association With Antigen‐Stimulated IL‐10 Production and Salivary IgA Secretion.” Scandinavian Journal of Medicine & Science in Sports 22, no. 3: 410–417. 10.1111/j.1600-0838.2010.01272.x. 21385218

[acel70182-bib-0032] Goldsmith, C. D. , T. Donovan , N. Vlahovich , and D. B. Pyne . 2021. “Unlocking the Role of Exercise on CD4+ T Cell Plasticity.” Frontiers in Immunology 12: 729366. 10.3389/fimmu.2021.729366. 34759918 PMC8573256

[acel70182-bib-0033] Graham, Z. A. , K. M. Lavin , S. M. O'Bryan , et al. 2021. “Mechanisms of Exercise as a Preventative Measure to Muscle Wasting.” American Journal of Physiology‐Cell Physiology 321, no. 1: C40–C57. 10.1152/ajpcell.00056.2021. 33950699 PMC8424676

[acel70182-bib-0034] Guilherme, J. P. L. F. , A. C. C. Tritto , K. N. North , A. H. Lancha Junior , and G. G. Artioli . 2014. “Genetics and Sport Performance: Current Challenges and Directions to the Future.” Revista Brasileira de Educação Física e Esporte 28: 177–193.

[acel70182-bib-0035] Haller, N. , M. Behringer , T. Reichel , et al. 2023. “Blood‐Based Biomarkers for Managing Workload in Athletes: Considerations and Recommendations for Evidence‐Based Use of Established Biomarkers.” Sports Medicine 53, no. 7: 1315–1333. 10.1007/s40279-023-01836-x. 37204619 PMC10197055

[acel70182-bib-0036] Haupt, S. , T. Niedrist , H. Sourij , S. Schwarzinger , and O. Moser . 2022. “The Impact of Exercise on Telomere Length, DNA Methylation and Metabolic Footprints.” Cells 11, no. 1: 153. 10.3390/cells11010153. 35011715 PMC8750279

[acel70182-bib-0037] Hernandez Cordero, A. I. , C. Peters , X. Li , et al. 2024. “Younger Epigenetic Age Is Associated With Higher Cardiorespiratory Fitness in Individuals With Airflow Limitation.” IScience 27, no. 10: 110934. 10.1016/j.isci.2024.110934. 39391738 PMC11465153

[acel70182-bib-0038] Hillary, R. F. , A. J. Stevenson , S. R. Cox , et al. 2021. “An Epigenetic Predictor of Death Captures Multi‐Modal Measures of Brain Health.” Molecular Psychiatry 26, no. 8: 3806–3816. 10.1038/s41380-019-0616-9. 31796892 PMC8550950

[acel70182-bib-0039] Hillary, R. F. , A. J. Stevenson , D. L. McCartney , et al. 2020. “Epigenetic Measures of Ageing Predict the Prevalence and Incidence of Leading Causes of Death and Disease Burden.” Clinical Epigenetics 12, no. 1: 115. 10.1186/s13148-020-00905-6. 32736664 PMC7394682

[acel70182-bib-0040] Horvath, S. 2013. “DNA Methylation Age of Human Tissues and Cell Types.” Genome Biology 14, no. 10: R115. 10.1186/gb-2013-14-10-r115. 24138928 PMC4015143

[acel70182-bib-0041] Horvath, S. , and A. J. Levine . 2015. “HIV‐1 Infection Accelerates Age According to the Epigenetic Clock.” Journal of Infectious Diseases 212, no. 10: 1563–1573. 10.1093/infdis/jiv277. 25969563 PMC4621253

[acel70182-bib-0042] Horvath, S. , J. Oshima , G. M. Martin , et al. 2018. “Epigenetic Clock for Skin and Blood Cells Applied to Hutchinson Gilford Progeria Syndrome and *Ex Vivo* Studies.” Aging (Albany NY) 10, no. 7: 1758–1775. 10.18632/aging.101508. 30048243 PMC6075434

[acel70182-bib-0044] Houseman, E. , W. Accomando , D. Koestler , et al. 2012. “DNA Methylation Arrays as Surrogate Measures of Cell Mixture Distribution.” BMC Bioinformatics 13: 86. 22568884 10.1186/1471-2105-13-86 PMC3532182

[acel70182-bib-0045] Hunt, R. M. , M. T. Elzayat , M. M. Markofski , M. Laughlin , and E. C. LaVoy . 2023. “Characterization of Transitional Memory CD4^+^ and CD8^+^ T‐Cell Mobilization During and After an Acute Bout of Exercise.” Frontiers in Sports and Active Living 5: 1120454. 10.3389/fspor.2023.1120454. 37139298 PMC10149718

[acel70182-bib-0046] Hunter, D. J. , L. S. James , B. Hussey , R. A. Ferguson , M. R. Lindley , and S. S. Mastana . 2023. “Impacts of Eccentric Resistance Exercise on DNA Methylation of Candidate Genes for Inflammatory Cytokines in Skeletal Muscle and Leukocytes of Healthy Males.” Genes 14, no. 2: 478. 10.3390/genes14020478. 36833405 PMC9957508

[acel70182-bib-0047] Jajtner, A. R. , J. R. Hoffman , J. R. Townsend , et al. 2016. “The Effect of Polyphenols on Cytokine and Granulocyte Response to Resistance Exercise.” Physiological Reports 4, no. 24: e13058. 10.14814/phy2.13058. 28039406 PMC5210375

[acel70182-bib-0048] Jokai, M. , F. Torma , K. M. McGreevy , et al. 2023. “DNA Methylation Clock DNAmFitAge Shows Regular Exercise Is Associated With Slower Aging and Systemic Adaptation.” GeroScience 45: 2805–2817. 10.1007/s11357-023-00826-1. 37209203 PMC10643800

[acel70182-bib-0115] Kasapis, C. , and P. D. Thompson . 2005. “The Effects of Physical Activity on Serum C‐Reactive Protein and Inflammatory Markers: A Systematic Review.” Journal of the American College of Cardiology 45, no. 10: 1563–1569. 10.1016/j.jacc.2004.12.077. Epub 2005 Apr 25. 15893167

[acel70182-bib-0051] Kostrzewa‐Nowak, D. , R. Nowak , T. Chamera , R. Buryta , W. Moska , and P. Cięszczyk . 2015. “Post‐Effort Chances in C‐Reactive Protein Level Among Soccer Players at the End of the Training Season.” Journal of Strength and Conditioning Research 29, no. 5: 1399–1405. 10.1519/JSC.0000000000000753. 25426511

[acel70182-bib-0119] Kraemer, W. J. , S. J. Fleck , and W. J. Evans . 1996. “Strength and Power Training: Physiological Mechanisms of Adaptation.” Exercise and Sport Sciences Reviews 24: 363–397. 8744256

[acel70182-bib-0052] Krüger, K. , S. Agnischock , A. Lechtermann , et al. 1985. “Intensive Resistance Exercise Induces Lymphocyte Apoptosis via Cortisol and Glucocorticoid Receptor‐Dependent Pathways.” Journal of Applied Physiology 110, no. 5: 1226–1232. 10.1152/japplphysiol.01295.2010. 21393471

[acel70182-bib-0053] Kusters, C. D. J. , and S. Horvath . 2025. “Quantification of Epigenetic Aging in Public Health.” Annual Review of Public Health 46, no. 1: 91–110. 10.1146/annurev-publhealth-060222-015657. PMC12180540 39681336

[acel70182-bib-0054] Kuznetsova, A. , P. B. Brockhoff , and R. H. B. Christensen . 2017. “lmerTest Package: Tests in Linear Mixed Effects Models.” Journal of Statistical Software 82, no. 13: 1–26. 10.18637/jss.v082.i13.

[acel70182-bib-0056] Levine, M. E. , A. T. Lu , A. Quach , et al. 2018. “An Epigenetic Biomarker of Aging for Lifespan and Healthspan.” Aging (Albany NY) 10, no. 4: 573–591. 10.18632/aging.101414. 29676998 PMC5940111

[acel70182-bib-0057] Li, Y. , S. Cui , W. Shi , et al. 2020. “Differential Placental Methylation in Preeclampsia, Preterm and Term Pregnancies.” Placenta 93: 56–63. 10.1016/j.placenta.2020.02.009. 32250740

[acel70182-bib-0058] Lightfoot, A. P. , A. McArdle , M. J. Jackson , and R. G. Cooper . 2015. “In the Idiopathic Inflammatory Myopathies (IIM), do Reactive Oxygen Species (ROS) Contribute to Muscle Weakness?” Annals of the Rheumatic Diseases 74, no. 7: 1340–1346. 10.1136/annrheumdis-2014-207172. 26063809

[acel70182-bib-0061] Loh, K. P. , C. Sanapala , M. Jensen‐Battaglia , et al. 2023. “Exercise and Epigenetic Ages in Older Adults With Myeloid Malignancies.” European Journal of Medical Research 28, no. 1: 180. 10.1186/s40001-023-01145-z. 37254221 PMC10227405

[acel70182-bib-0062] Lu, A. T. , A. M. Binder , J. Zhang , et al. 2022. “DNA Methylation GrimAge Version 2.” Aging (Albany NY) 14, no. 23: 9484–9549. 10.18632/aging.204434. 36516495 PMC9792204

[acel70182-bib-0063] Lu, A. T. , A. Quach , J. G. Wilson , et al. 2019. “DNA Methylation GrimAge Strongly Predicts Lifespan and Healthspan.” Aging (Albany NY) 11, no. 2: 303–327. 10.18632/aging.101684. 30669119 PMC6366976

[acel70182-bib-0064] Lundin, J. I. , U. Peters , Y. Hu , et al. 2024. “Methylation Patterns Associated With C‐Reactive Protein in Racially and Ethnically Diverse Populations.” Epigenetics 19, no. 1: 2333668. 10.1080/15592294.2024.2333668. 38571307 PMC10996836

[acel70182-bib-0066] Markov, A. , J. Bussweiler , N. Helm , et al. 2023. “Acute Effects of Concurrent Muscle Power and Sport‐Specific Endurance Exercises on Markers of Immunological Stress Response and Measures of Muscular Fitness in Highly Trained Youth Male Athletes.” European Journal of Applied Physiology 123, no. 5: 1015–1026. 10.1007/s00421-022-05126-8. 36624248 PMC9829527

[acel70182-bib-0067] Marqués‐Jiménez, D. , J. Calleja‐González , I. Arratibel , A. Delextrat , and N. Terrados . 2016. “Are Compression Garments Effective for the Recovery of Exercise‐Induced Muscle Damage? A Systematic Review With Meta‐Analysis.” Physiology & Behavior 153: 133–148. 10.1016/j.physbeh.2015.10.027. 26522739

[acel70182-bib-0068] McCrory, C. , G. Fiorito , B. Hernandez , et al. 2021. “GrimAge Outperforms Other Epigenetic Clocks in the Prediction of Age‐Related Clinical Phenotypes and All‐Cause Mortality.” Journals of Gerontology. Series A, Biological Sciences and Medical Sciences 76, no. 5: 741–749. 10.1093/gerona/glaa286. 33211845 PMC8087266

[acel70182-bib-0069] McGee, S. L. , and M. Hargreaves . 2019. “Epigenetics and Exercise.” Trends in Endocrinology and Metabolism 30, no. 9: 636–645. 10.1016/j.tem.2019.06.002. 31279665

[acel70182-bib-0070] McGreevy, K. M. , Z. Radak , F. Torma , et al. 2023. “DNAmFitAge: Biological Age Indicator Incorporating Physical Fitness.” Aging (Albany NY) 15, no. 10: 3904–3938. 10.18632/aging.204538. 36812475 PMC10258016

[acel70182-bib-0071] Mendham, A. E. , C. E. Donges , E. A. Liberts , and R. Duffield . 2011. “Effects of Mode and Intensity on the Acute Exercise‐Induced IL‐6 and CRP Responses in a Sedentary, Overweight Population.” European Journal of Applied Physiology 111, no. 6: 1035–1045. 10.1007/s00421-010-1724-z. 21088973

[acel70182-bib-0072] Middleton, L. Y. M. , J. Dou , J. Fisher , et al. 2022. “Saliva Cell Type DNA Methylation Reference Panel for Epidemiological Studies in Children.” Epigenetics 17, no. 2: 161–177. 10.1080/15592294.2021.1890874. 33588693 PMC8865319

[acel70182-bib-0073] Moghadam‐Kia, S. , C. V. Oddis , and R. Aggarwal . 2016. “Approach to Asymptomatic Creatine Kinase Elevation.” Cleveland Clinic Journal of Medicine 83, no. 1: 37–42. 10.3949/ccjm.83a.14120. 26760521 PMC4871266

[acel70182-bib-0074] Morgado, J. P. , C. N. Matias , J. F. Reis , D. Curto , F. B. Alves , and C. P. Monteiro . 2020. “The Cellular Composition of the Innate and Adaptive Immune System Is Changed in Blood in Response to Long‐Term Swimming Training.” Frontiers in Physiology 11: 471. 10.3389/fphys.2020.00471. 32477166 PMC7235416

[acel70182-bib-0075] Mougios, V. 2007. “Reference Intervals for Serum Creatine Kinase in Athletes.” British Journal of Sports Medicine 41, no. 10: 674–678. 10.1136/bjsm.2006.034041. 17526622 PMC2465154

[acel70182-bib-0076] Murtagh, E. M. , C. Boreham , A. Nevill , et al. 2005. “Acute Responses of Inflammatory Markers of Cardiovascular Disease Risk to a Single Walking Session.” Journal of Physical Activity and Health 2, no. 3: 324–332.

[acel70182-bib-0077] Nagata, M. , S. Komaki , Y. Nishida , et al. 2024. “Influence of Physical Activity on the Epigenetic Clock: Evidence From a Japanese Cross‐Sectional Study.” Clinical Epigenetics 16, no. 1: 142. 10.1186/s13148-024-01756-1. 39407257 PMC11481432

[acel70182-bib-0120] Nieman, D. C. , K. S. Buckley , D. A. Henson , et al. 1995. “Immune Function in Marathon Runners Versus Sedentary Controls.” Medicine and Science in Sports and Exercise 27, no. 7: 986–992. 10.1249/00005768-199507000-00006. 7564985

[acel70182-bib-0078] Nieman, D. C. , and B. K. Pedersen . 1999. “Exercise and Immune Function. Recent Developments.” Sports Medicine 27, no. 2: 73–80. 10.2165/00007256-199927020-00001. 10091272

[acel70182-bib-0079] Nitert, M. D. , T. Dayeh , P. Volkov , et al. 2012. “Impact of an Exercise Intervention on DNA Methylation in Skeletal Muscle From First‐Degree Relatives of Patients With Type 2 Diabetes.” Diabetes 61, no. 12: 3322–3332. 10.2337/db11-1653. 23028138 PMC3501844

[acel70182-bib-0116] Ntovas, P. , N. Loumprinis , P. Maniatakos , L. Margaritidi , and C. Rahiotis . 2022. “The Effects of Physical Exercise on Saliva Composition: A Comprehensive Review.” Dentistry Journal (Basel) 10, no. 1: 7. 10.3390/dj10010007. PMC8775020 35049605

[acel70182-bib-0080] Pandorf, C. E. , F. Haddad , C. Wright , P. W. Bodell , and K. M. Baldwin . 2009. “Differential Epigenetic Modifications of Histones at the Myosin Heavy Chain Genes in Fast and Slow Skeletal Muscle Fibers and in Response to Muscle Unloading.” American Journal of Physiology. Cell Physiology 297, no. 1: C6–C16. 10.1152/ajpcell.00075.2009. 19369448 PMC2711647

[acel70182-bib-0114] Pfab, F. , J. Sieland , C. Haser , W. Banzer , and T. Kocher . 2023. “Genetische Faktoren bei Muskelverletzungen im Sport [Genetics in Sports‐Muscle Injuries].” Orthopadie (Heidelb) 52, no. 11: 889–896. German. doi:. 10.1007/s00132-023-04439-6. Epub 2023 Sep 29. 37773215

[acel70182-bib-0081] Plagg, B. , and S. Zerbe . 2021. “How Does the Environment Affect Human Ageing? An Interdisciplinary Review.” Journal of Gerontology and Geriatrics 69: 53–67. 10.36150/2499-6564-420.

[acel70182-bib-0082] Potempa, M. , P. C. Hart , I. M. Rajab , and L. A. Potempa . 2025. “Redefining CRP in Tissue Injury and Repair: More Than an Acute Pro‐Inflammatory Mediator.” Frontiers in Immunology 16: 1564607. 10.3389/fimmu.2025.1564607. 40093010 PMC11906453

[acel70182-bib-0083] Potthoff, M. J. , H. Wu , M. A. Arnold , et al. 2007. “Histone Deacetylase Degradation and MEF2 Activation Promote the Formation of Slow‐Twitch Myofibers.” Journal of Clinical Investigation 117, no. 9: 2459–2467. 10.1172/JCI31960. 17786239 PMC1957540

[acel70182-bib-0084] Puzianowska‐Kuźnicka, M. , M. Owczarz , K. Wieczorowska‐Tobis , et al. 2016. “Interleukin‐6 and C‐Reactive Protein, Successful Aging, and Mortality: The PolSenior Study.” Immunity & Ageing 13, no. 1: 21. 10.1186/s12979-016-0076-x. 27274758 PMC4891873

[acel70182-bib-0085] Radák, Z. , D. Aczél , I. Fejes , et al. 2025. “Slowed Epigenetic Aging in Olympic Champions Compared to Non‐Champions.” Geroscience 47, no. 2: 2555–2565. 10.1007/s11357-024-01440-5. 39601999 PMC11978583

[acel70182-bib-0087] Romagnoli, M. , F. Sanchis‐Gomar , R. Alis , et al. 2016. “Changes in Muscle Damage, Inflammation, and Fatigue‐Related Parameters in Young Elite Soccer Players After a Match.” Journal of Sports Medicine and Physical Fitness 56, no. 10: 1198–1205. 26558831

[acel70182-bib-0088] Rönn, T. , P. Volkov , C. Davegårdh , et al. 2013. “A Six Months Exercise Intervention Influences the Genome‐Wide DNA Methylation Pattern in Human Adipose Tissue.” PLoS Genetics 9, no. 6: e1003572. 10.1371/journal.pgen.1003572. 23825961 PMC3694844

[acel70182-bib-0089] Ryan, J. L. , E. E. Pracht , and B. L. Orban . 2019. “Inpatient and Emergency Department Costs From Sports Injuries Among Youth Aged 5‐18 Years.” BMJ Open Sport & Exercise Medicine 5, no. 1: e000491. 10.1136/bmjsem-2018-000491. PMC6539161 31191961

[acel70182-bib-0090] Saidi, K. , A. B. Abderrahman , I. Laher , et al. 2024. “Immune Inflammation Markers and Physical Fitness During a Congested Match Play Period in Elite Male Soccer Players.” Scientific Reports 14, no. 1: 30312. 10.1038/s41598-024-81225-0. 39639055 PMC11621450

[acel70182-bib-0092] Schild, M. , G. Eichner , T. Beiter , et al. 2016. “Effects of Acute Endurance Exercise on Plasma Protein Profiles of Endurance‐Trained and Untrained Individuals Over Time.” Mediators of Inflammation 2016: 4851935. 10.1155/2016/4851935. 27239103 PMC4867072

[acel70182-bib-0093] Seaborne, R. A. , J. Strauss , M. Cocks , et al. 2018. “Human Skeletal Muscle Possesses an Epigenetic Memory of Hypertrophy.” Scientific Reports 8, no. 1: 1898. 10.1038/s41598-018-20287-3. 29382913 PMC5789890

[acel70182-bib-0094] Seki, Y. , D. Aczel , F. Torma , et al. 2023. “No Strong Association Among Epigenetic Modifications by DNA Methylation, Telomere Length, and Physical Fitness in Biological Aging.” Biogerontology 24, no. 2: 245–255. 10.1007/s10522-022-10011-0. 36592269 PMC10006047

[acel70182-bib-0095] Silva, A. F. , F. T. González‐Fernández , H. I. Ceylan , et al. 2022. “Relationships Between Fitness Status and Blood Biomarkers in Professional Soccer Players.” Journal of Healthcare Engineering 2022: 5135817. 10.1155/2022/5135817. 35449856 PMC9017447

[acel70182-bib-0121] Simonson, S. R. , and C. G. Jackson . 2004. “Leukocytosis Occurs in Response to Resistance Exercise in Men.” Journal of Strength and Conditioning Research 18, no. 2: 266–271. 10.1519/R-12572.1. 15142013

[acel70182-bib-0096] Souglis, A. G. , A. Papapanagiotou , G. C. Bogdanis , A. K. Travlos , N. G. Apostolidis , and N. D. Geladas . 2015. “Comparison of Inflammatory Responses to a Soccer Match Between Elite Male and Female Players.” Journal of Strength and Conditioning Research 29, no. 5: 1227–1233. 10.1519/JSC.0000000000000767. 25436628

[acel70182-bib-0097] Spielmann, G. , B. K. McFarlin , D. P. O'Connor , P. J. Smith , H. Pircher , and R. J. Simpson . 2011. “Aerobic Fitness Is Associated With Lower Proportions of Senescent Blood T‐Cells in Man.” Brain, Behavior, and Immunity 25, no. 8: 1521–1529. 10.1016/j.bbi.2011.07.226. 21784146

[acel70182-bib-0098] Spólnicka, M. , E. Pośpiech , J. G. Adamczyk , et al. 2018. “Modified Aging of Elite Athletes Revealed by Analysis of Epigenetic Age Markers.” Aging (Albany NY) 10, no. 2: 241–252. 10.18632/aging.101385. 29466246 PMC5842850

[acel70182-bib-0099] Suzuki, K. , S. Nakaji , M. Yamada , M. Totsuka , K. Sato , and K. Sugawara . 2002. “Systemic Inflammatory Response to Exhaustive Exercise. Cytokine Kinetics.” Exercise Immunology Review 8: 6–48. 12690937

[acel70182-bib-0100] Tarnowski, M. , P. Tomasiak , M. Tkacz , K. Zgutka , and K. Piotrowska . 2022. “Epigenetic Alterations in Sports‐Related Injuries.” Genes 13, no. 8: 1471. 10.3390/genes13081471. 36011382 PMC9408207

[acel70182-bib-0101] Tay, J. H. , Y. E. Chew , W. Wang , et al. 2025. “DNAm Age Differences Between Infinium methylationEPICv1 vs EPICv2 in Buffy Coat, PBMC, and Saliva Samples.” Communications Biology 8, no. 1: 654. 10.1038/s42003-025-08021-y. 40269264 PMC12019316

[acel70182-bib-0102] Thorpe, R. , and C. Sunderland . 2012. “Muscle Damage, Endocrine, and Immune Marker Response to a Soccer Match.” Journal of Strength and Conditioning Research 26, no. 10: 2783–2790. 10.1519/JSC.0b013e318241e174. 22124357

[acel70182-bib-0103] Trentacosta, N. 2020. “Pediatric Sports Injuries.” Pediatric Clinics of North America 67, no. 1: 205–225. 10.1016/j.pcl.2019.09.013. 31779833

[acel70182-bib-0104] Varillas‐Delgado, D. , J. Del Coso , J. Gutiérrez‐Hellín , et al. 2022. “Genetics and Sports Performance: The Present and Future in the Identification of Talent for Sports Based on DNA Testing.” European Journal of Applied Physiology 122, no. 8: 1811–1830. 10.1007/s00421-022-04945-z. 35428907 PMC9012664

[acel70182-bib-0105] Varillas‐Delgado, D. , J. Gutierrez‐Hellín , and A. Maestro . 2023. “Genetic Profile in Genes Associated With Sports Injuries in Elite Endurance Athletes.” International Journal of Sports Medicine 44, no. 1: 64–71. 10.1055/a-1917-9212. 35921847

[acel70182-bib-0106] Velissaris, D. , N. Pantzaris , I. Koniari , et al. 2017. “C‐Reactive Protein and Frailty in the Elderly: A Literature Review.” Journal of Clinical Medical Research 9, no. 6: 461–465. 10.14740/jocmr2959w. PMC5412518 28496545

[acel70182-bib-0107] Voisin, S. , N. Eynon , X. Yan , and D. J. Bishop . 2015. “Exercise Training and DNA Methylation in Humans.” Acta Physiologica (Oxford, England) 213, no. 1: 39–59. 10.1111/apha.12414. 25345837

[acel70182-bib-0108] Wallace, J. L. , and K. I. Norton . 2014. “Evolution of World Cup Soccer Final Games 1966‐2010: Game Structure, Speed and Play Patterns.” Journal of Science and Medicine in Sport 17, no. 2: 223–228. 10.1016/j.jsams.2013.03.016. 23643671

[acel70182-bib-0110] Wielscher, M. , P. R. Mandaviya , B. Kuehnel , et al. 2022. “DNA Methylation Signature of Chronic Low‐Grade Inflammation and Its Role in Cardio‐Respiratory Diseases.” Nature Communications 13, no. 1: 2408. 10.1038/s41467-022-29792-6. PMC9065016 35504910

[acel70182-bib-0111] Yparraguirre Salcedo, K. G. , A. B. Rivera Prado , L. Lloja Lozano , V. F. Chambilla Quispe , and C. W. Ramirez Atencio . 2024. “Evaluating Muscle Damage Biomarkers in Adolescent Athletes: Implications for Public Health in Tacna, Peru‐2023.” International Journal of Environmental Research and Public Health 21, no. 11: 1394. 10.3390/ijerph21111394. 39595661 PMC11593879

[acel70182-bib-0112] Zannas, A. S. , J. Arloth , T. Carrillo‐Roa , et al. 2015. “Lifetime Stress Accelerates Epigenetic Aging in an Urban, African American Cohort: Relevance of Glucocorticoid Signaling.” Genome Biology 16: 266. 10.1186/s13059-015-0828-5. 26673150 PMC4699359

[acel70182-bib-0113] Zheng, X. , X. Liu , Y. Guo , et al. 2025. “Physical Exercise and Epigenetic Modifications in Skeletal Muscle, Brain, and Heart.” Epigenetics & Chromatin 18, no. 1: 12. 10.1186/s13072-025-00576-8. 40114219 PMC11927307

