# Supplementary material for: Epigenetic Age Monitoring in Professional Soccer Players for Tracking Recovery and the Effects of Strenuous Exercise
Source: Aging Cell. 2025 Jul 28;24(10):e70182. doi: 10.1111/acel.70182 (PMC12507406; doi:10.1111/acel.70182)

Supplementary Information


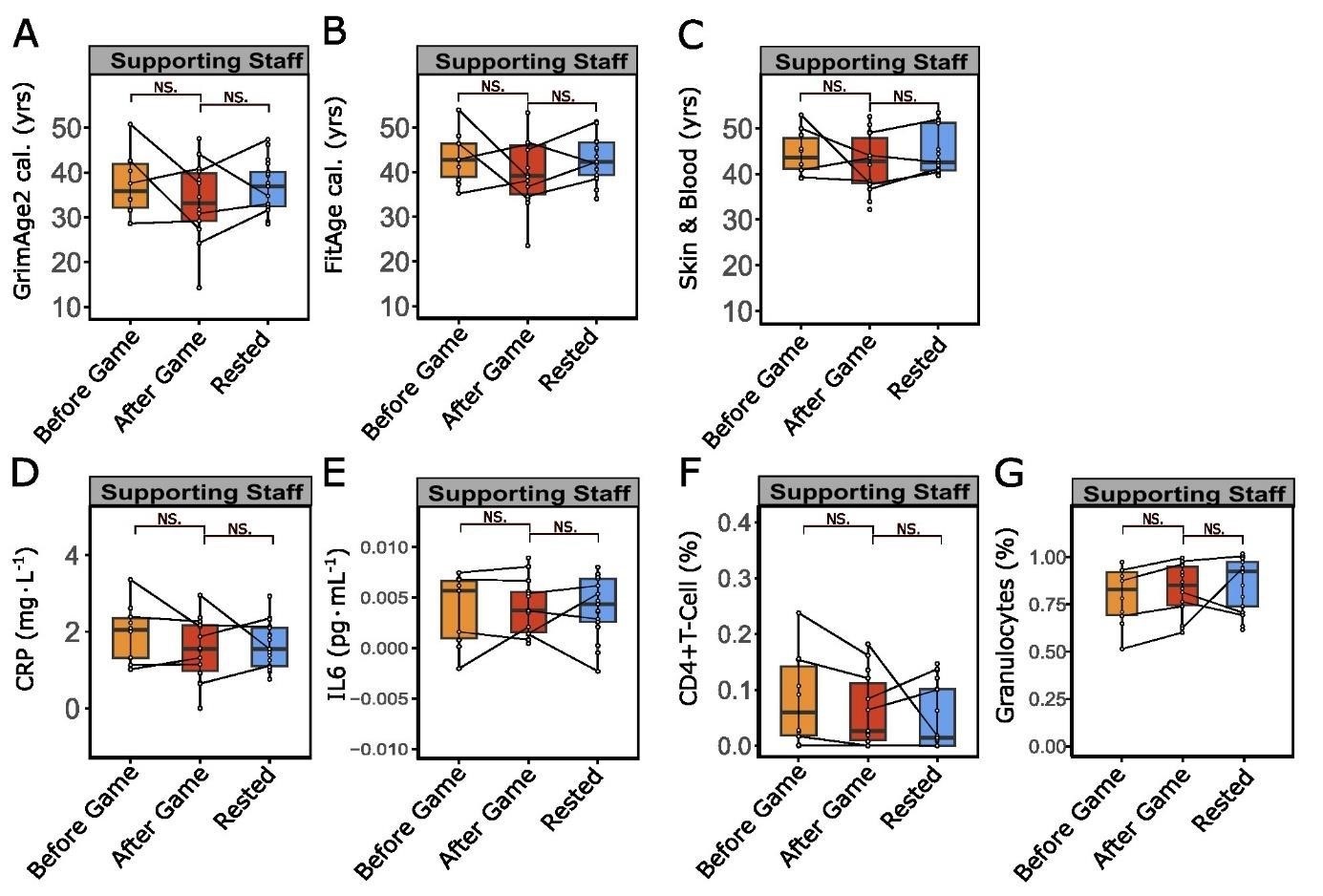


**Supplementary Figure 1: Changes in biological age predictors and immunological events upon intense physical activity in supporting staff members.** **(A-C)** Epigenetic profiles (DNAm) of saliva samples collected from supporting staff members (n = 4) at resting states (before game, rested) or immediately after intensive physical activity (after game) were used to estimate DNAm-based biological aging clocks. **(A) GrimAge2 cal.** (before vs. after game p = 0.265; after game vs. rested p = 0.298), **(B) FitAge cal.** (before vs. after game p = 0.242; after game to rested p = 0.328) and **(C)** chronological age predictor **Skin & Blood Clock** (before vs. after game p = 0.480; after game vs. rested p = 0.299). (D-G) Boxplots illustrate changes of DNAm-derived surrogate blood protein levels before and after physical activity (before/after game) and after recovery (after rest) for inflammation markers **(D) Methylation-based estimator of CRP** (before vs. after game p = 0.227, β = 0.27582, 95% CI: [-0.1576053, 0.7066444], f² = 0.1428891; after game vs. rested p = 0.791, β = -0.05611, 95% CI: [-0.4674867, 0.3455589], f² = 0.1428891) and **(E) Methylation-based estimator of IL-6** (before vs. after game p = 0.739, β = -0.0003105, 95% CI: [-0.0020884276, 0.0014753087], f² = 0.004445367; after game vs. rested p = 0.5169, β = -0.0005695, 95% CI: [-0.0022283282, 0.0011238408], f² = 0.004445367) as well as immune cell activity for **(F)** **CD4+T-Cells** (before vs. after game p = 0.280, β = 0.019986, 95% CI: [-0.015234103, 0.05506440], f² = 0.02160369; after game vs. rested p = 0.755, β = -0.005382, 95% CI: [-0.038703700, 0.02739882], f² = 0.02160369) and **(G) Granulocytes** (before vs. after game p = 0.358, β = -0.0370919, 95% CI: [-0.11370671, 0.03980036], f² = 0.01600528; after game vs. rested p = 0.709, β = 0.0140510, 95% CI: [-0.05751153, 0.08684130], f² = 0.01600528). (A-G) Each dot represents one sample from one participant, samples from the same participant are connected by line across physical activity groups, significant changes (p-values) were tested using a linear mixed effect model with chronological age, timepoint (before game, after game or rested) and batch number as fixed and player id as random effect. Plots show median (bold line) with interquartile range (box) and 1.5 fold interquartile range (whiskers). Significance levels are indicated by * (p <= 0.05), ** (p <= 0.01) and NS. (p > 0.05). Cal.: GrimAge2 and FitAge predictions were calibrated to the actual age range of players.


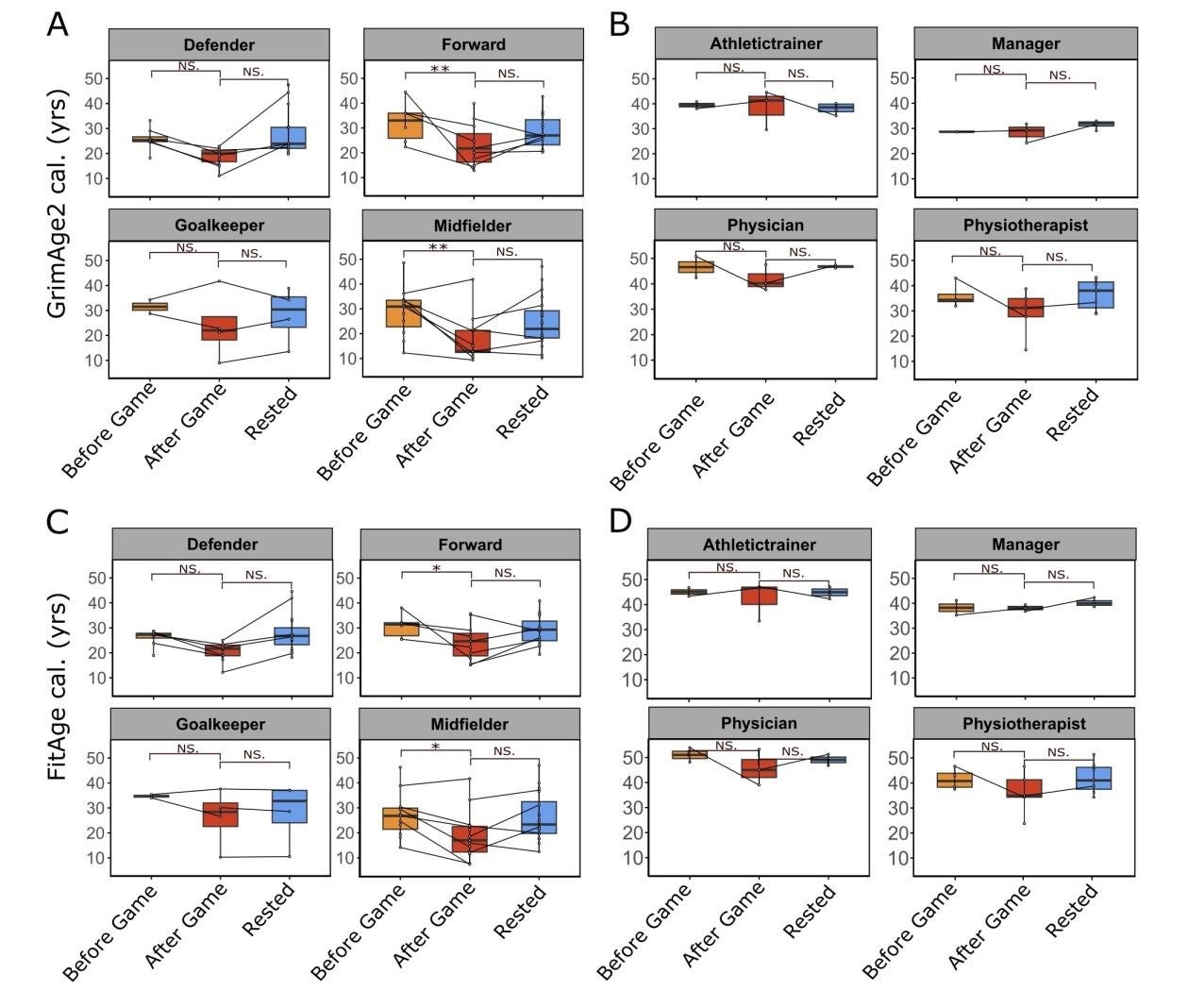


**Supplementary Figure 2: Instantaneous effects of high physical load on DNAm-based age predictors** **in athlete and supporting staff subgroups.** **(A-D)** Epigenetic profiles of saliva samples collected from athletes (n < 19) at resting states (before game, rested) or immediately after intensive physical activity (after game) were used to estimate DNAm-based biological aging clocks. **(A) GrimAge2 cal. for athletes** playing different positions (before vs. after game: Defender, β = -6.65, 95% CI [-12.54, -0.94], f² = 0.34, p = 0.074 | Forward, β = -10.67, 95% CI [-16.76, -3.89], f² = 0.26, p = 0.00791 | Goalkeeper, β = -1.39, 95% CI [-8.33, 5.96], f² = 0.62, p = 0.644 | Midfielder, β = -10.00, 95% CI [-15.54, -4.46], f² = 1.25, p = 0.00414 ; after game vs. rested: Defender, β = -8.80, 95% CI [-16.01, -1.59], f² = 0.34, p = 0.585 | Forward, β = -5.98, 95% CI [-13.80, 1.84], f² = 0.26, p = 0.130 | Goalkeeper, β = -1.78, 95% CI [-8.55, 5.00], f² = 0.87, p = 0.970 | Midfielder, β = -5.99, 95% CI [-13.18, 1.16], f² = 1.25, p = 0.0966); **(B) GrimAge2 cal. for Supporting Staff** members (before vs. after game: Athletictrainer, β = -0.20, 95% CI [-8.85, 8.44], f² = 0.41, p = 0.954 | Manager, β = 0.27, 95% CI [-6.19, 6.73], f² = 0.30, p = 0.9187 | Physician, β = -3.40, 95% CI [-18.31, 11.50], f² = 0.30, p = 0.520 | Physiotherapist, β = -5.29, 95% CI [-16.04, 5.47], f² = 0.21, p = 0.305; after game vs. rested: Athletictrainer, β = -2.40, 95% CI [-10.65, 5.86], f² = 0.41, p = 0.489 | Manager, β = 2.60, 95% CI [-3.34, 8.53], f² = 0.30, p = 0.312 | Physician, β = -1.14, 95% CI [-17.33, 15.04], f² = 0.30, p = 0.837 | Physiotherapist, β = 0.08, 95% CI [-9.65, 9.80], f² = 0.21, p = 0.987); **(C) FitAge cal. for athletes** at different positions (before vs. after game: Defender, β = -5.06, 95% CI [-9.06, -1.10], f² = 0.55, p = 0.0461 | Forward, β = -6.65, 95% CI [-12.00, -1.37], f² = 0.21, p = 0.0282 | Goalkeeper, β = -2.06, 95% CI [-6.22, 1.62], f² = 0.92, p = 0.449 | Midfielder, β = -4.91, 95% CI [-10.77, -0.07], f² = 1.95, p = 0.0110; after game vs. rested: Defender, β = -7.09, 95% CI [-11.67, -2.50], f² = 0.55, p = 0.410 | Forward, β = -4.27, 95% CI [-9.13, 2.78], f² = 0.21, p = 0.282 | Goalkeeper, β = -0.59, 95% CI [-2.91, 1.77], f² = 0.95, p = 0.532 | Midfielder, β = -4.07, 95% CI [-10.77, 2.67], f² = 1.95, p = 0.483)**; (D) FitAge cal. for Supporting Staff** members (before vs. after game: Athletictrainer, β = -1.79, 95% CI [-9.32, 5.74], f² = 0.54, p = 0.567 | Manager, β = -0.43, 95% CI [-6.20, 5.34], f² = 0.20, p = 0.856 | Physician, β = -3.48, 95% CI [-21.08, 14.13], f² = 0.25, p = 0.574 | Physiotherapist, β = -3.99, 95% CI [-14.13, 6.15], f² = 0.18, p = 0.408; after game vs. rested: Athletictrainer, β = -1.37, 95% CI [-8.56, 5.81], f² = 0.54, p = 0.644 | Manager, β = 2.19, 95% CI [-3.11, 7.50], f² = 0.20, p = 0.336 | Physician, β = -3.77, 95% CI [-22.88, 15.35], f² = 0.25, p = 0.575 | Physiotherapist, β = 0.05, 95% CI [-9.12, 9.22], f² = 0.18, p = 0.990).**(A–D)** Each dot represents one sample from one participant, samples from the same participants are connected by line across physical activity groups, significant changes (p-values) were tested using a linear mixed effect model with chronological age, timepoint (before game, after game or rested) and batch number as fixed and player ID as random effect. Plots show median (bold line) with interquartile range (box) and 1.5 fold interquartile range (whiskers). Significance levels are indicated by * (p <= 0.05), ** (p <= 0.01) and NS. (p > 0.05). Cal.: GrimAge2 and FitAge predictions were calibrated to the actual age range of players.


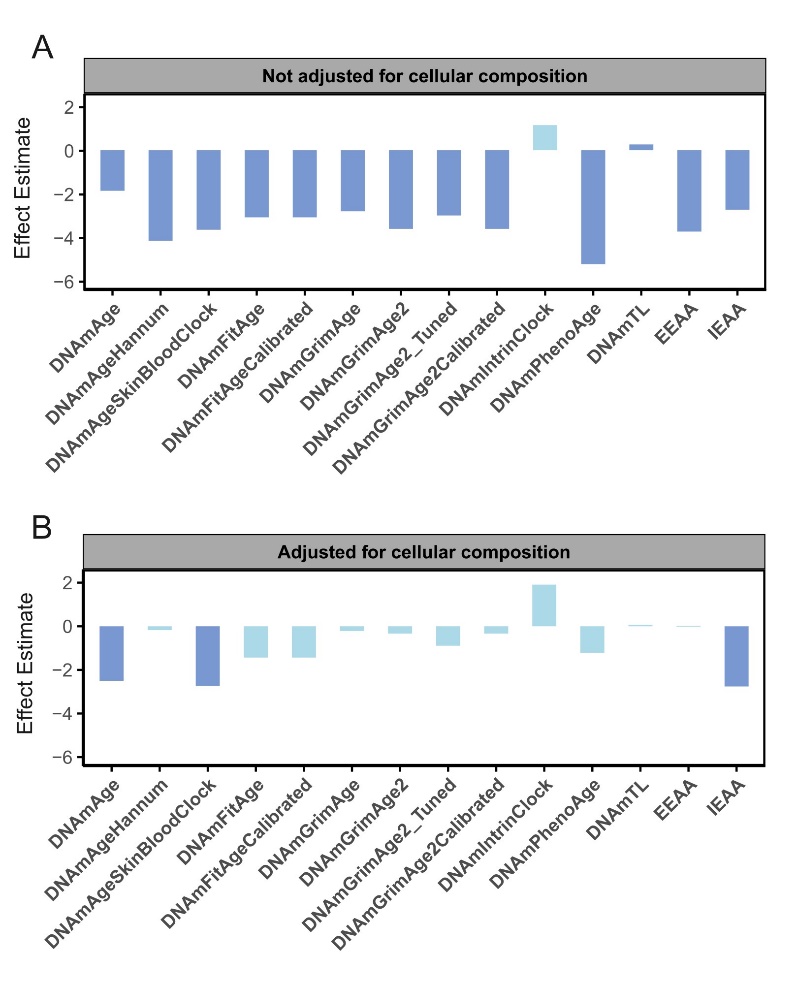


**Supplementary Figure 3: Epigenetic Clocks Before and After Adjustment for Immune Cell Composition.** (**A**) show estimated coefficients for each epigenetic clock based on a linear mixed-effects regression model, adjusting for chronological age, timepoint (before game vs. after game), and batch number as fixed effects, with player ID as a random effect. (**B**) includes additional adjustment for scaled proportions of CD4+ T cells and CD8+ CD28−CD45RA+ cells. Significance is indicated by color: dark blue (p ≤ 0.05) and light blue (p > 0.05).


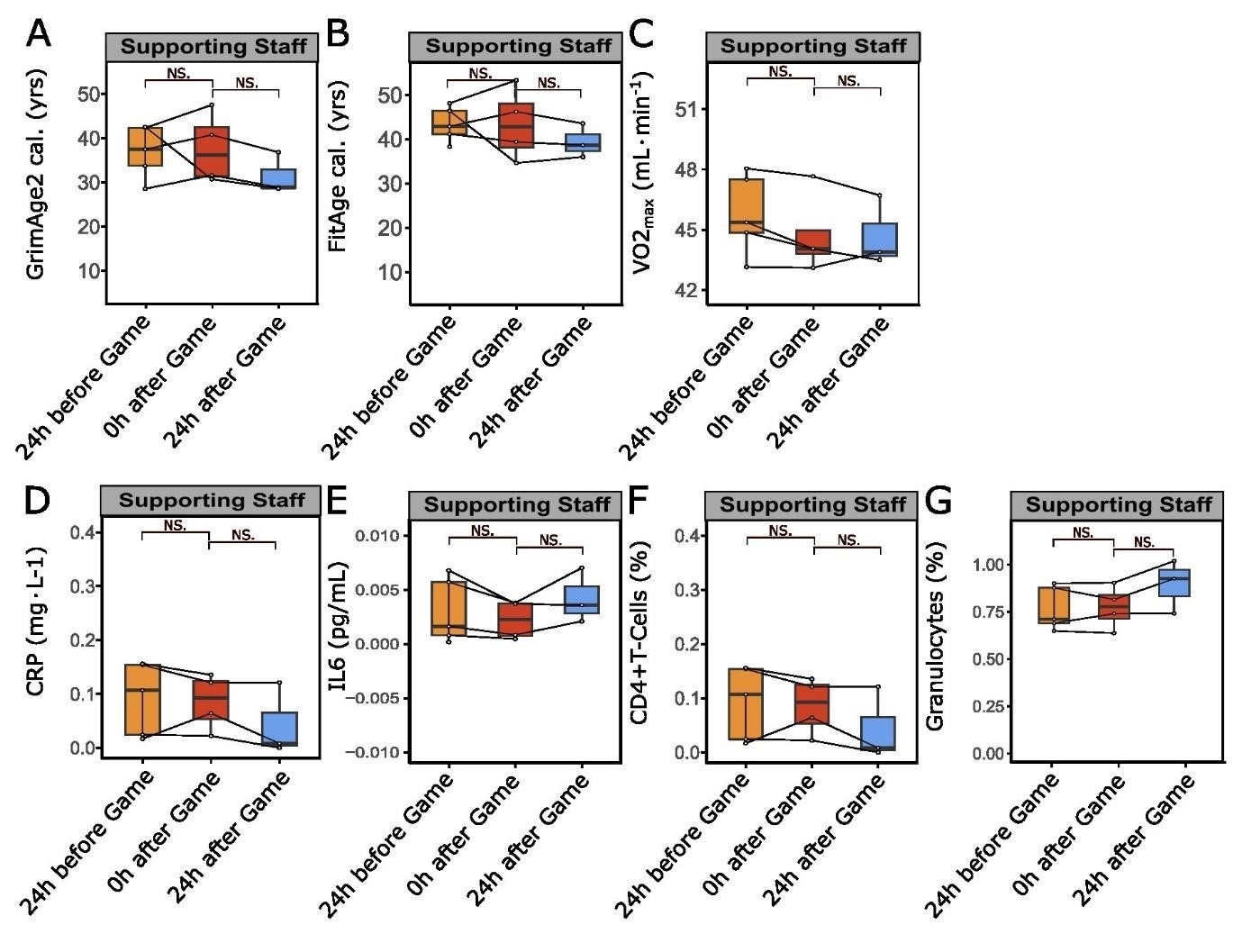


**Supplementary Figure 4: Instantaneous effects of high physical load on DNAm-derived immunological factors in supporting staff members**. **(A-G)** Epigenetic profiles (DNAm) of saliva samples from supporting staff (n = 4), collected as control, also during mid-season game (samples 3, 4 and 5) 24 hrs before (24 hrs before game) or immediately after the athletes underwent intensive physical activity (straight after game), were analyzed in addition to samples taken 24 hrs after the athletes high physical strain (24 hrs after game). The data was used to estimate DNAm-based biological aging clocks **(A) GrimAge2 cal.** (24hrs before vs. straight after game p = 0.944, β = 0.2573, 95% CI: [-6.027577, 6.503034], f² = 0.08350215; straight after vs. 24 hrs after game p = 0.337, β = -6.1591, 95% CI: [-17.250646, 3.986773], f² = 0.08350215) and **(B) FitAge cal.** (24hrs before vs. straight after game p = 0.663, β = 1.459, 95% CI: [-4.320914, 6.991435], f² = 0.06014719; straight after vs. 24 hrs after game p = 0.409, β = -4.682, 95% CI: [-14.389327, 4.437827], f² = 0.06014719). Analysis of DNAm-based endurance estimator **(C) VO2max** (24hrs before vs. straight after game p = 0.344, β = 0.4994, 95% CI: [-0.3456296, 1.3394471], f² = 0.03714855; straight after vs. 24 hrs after game p = 0.862, β = 0.1470, 95% CI: [-1.2518180, 1.6027626], f² = 0.03714855), plasma protein surrogate factors **(D) Methylation-based estimator of CRP** (24hrs before vs. straight after game p = 0.512, β = -0.2010, 95% CI: [-0.69995376, 0.3172509], f² = 0.1813306; straight after vs. 24 hrs after game p = 0.444, β = -0.3932, 95% CI: [-1.34162511, 0.4344582], f² = 0.1813306) and **(E) Methylation-based estimator of IL-6** (24hrs before vs. straight after game p = 0.754, β = 0.0005472, 95% CI: [-0.002585062, 0.003457823], f² = 0.06889536; straight after vs. 24 hrs after game p = 0.426, β = 0.0023169, 95% CI: [-0.002434549, 0.008064647], f² = 0.06889536) and immune cell type estimates for **(F) CD4+T-Cells** (24hrs before vs. straight after game p = 0.762, β = 0.007289, 95% CI: [-0.03253834, 0.04799062], f² = 0.06944642; straight after vs. 24 hrs after game p = 0.798, β = -0.010342, 95% CI: [-0.08207356, 0.05589386], f² = 0.06944642) and **(G) Granulocytes** (24hrs before vs. straight after game p = 0.971, β = -0.001608, 95% CI: [-0.07676044, 0.07239193], f² = 0.1321173; straight after vs. 24 hrs after game p = 0.623, β = 0.037183, 95% CI: [-0.08581749, 0.16810368], f² = 0.1321173). Each dot represents one sample from one participant, samples from the same participant are connected by line across physical activity groups, significant changes (p-values) were tested using a linear mixed effect model with chronological age, timepoint (24 hrs before, straight after or 24 hrs after game) and batch number as fixed and player id as random effect. Significance levels are indicated by * (p <= 0.05), ** (p <= 0.01) and NS. (p > 0.05). Cal.: GrimAge2 and FitAge predictions were calibrated to the actual age range of players.


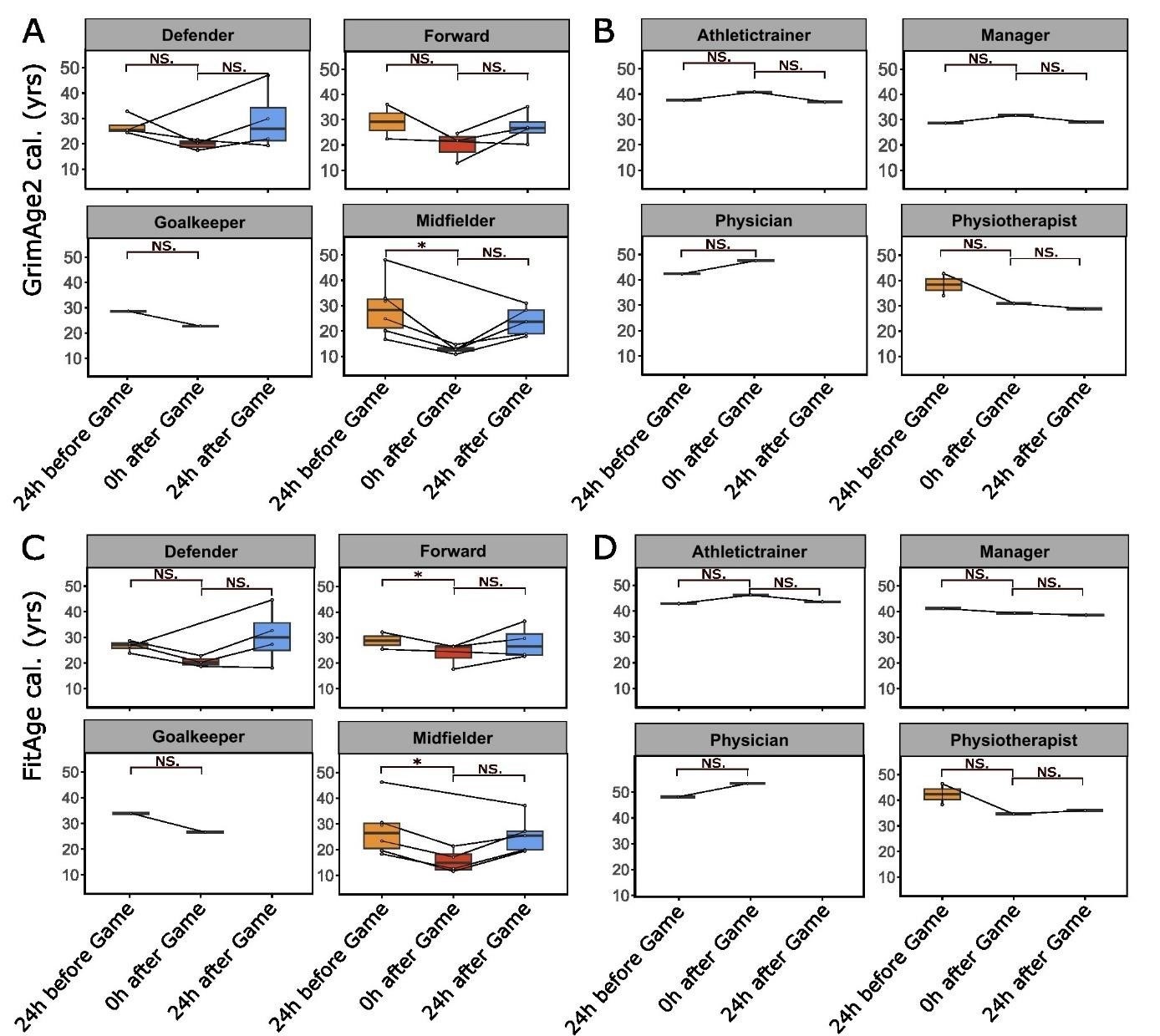


**Supplementary Figure 5: Instantaneous effects of high physical load on DNAm-based age predictors in athlete and supporting staff subgroups. (A-D)** Epigenetic profiles (DNAm) of saliva samples collected from athletes (n < 19) and supporting staff members (n = 4) at resting states (24 hrs before game, 24 hrs after game) or immediately after intensive physical activity (0h straight after game) were used to estimate DNAm-based biological aging clocks. **(A) GrimAge2 cal. for athletes** at different positions (24 hrs before vs. straight after game: Defender, β = -5.66, 95% CI [-17.53, 4.63], f² = 0.21, p = 0.38 | Forward, β = -14.13, 95% CI [-19.18, -4.79], f² = 0.81, p = 0.052 | Goalkeeper, p = 1.00 | Midfielder, β = -12.48, 95% CI [-18.38, -6.58], f² = 3.63, p = 0.012; straight after game vs. 24 hrs after game: Defender, p = 0.223 | Forward, p = 0.0776 | Goalkeeper, missing data | Midfielder, p = 0.130); **(B) GrimAge2 cal. for Supporting Staff** members (24 hrs before vs. straight after game: Athletictrainer, p = 1.00 | Manager, p = 1.00 | Physician, missing data | Physiotherapist, p = 0.505; straight after game vs. 24 hrs after game: Athletictrainer, p = 1.00 | Manager, p = 1.00 | Physician, missing data | Physiotherapist, p = 0.85; 24 hrs before game vs. 24 hrs after game: Physician, p = 1.00); **(C) FitAge cal. for athletes** at different positions (24 hrs before vs. straight after game: Defender, p = 0.387 | Forward, p = 0.0282 | Goalkeeper, p = 1.00 | Midfielder, p = 0.0114; straight after game vs. 24 hrs after game: Defender, p = 0.123 | Forward, p = 0.29 | Goalkeeper, missing data | Midfielder, p = 0.0941); **(D) FitAge cal. for Supporting Staff** members (24 hrs before vs. straight after game: Athletictrainer, p = 1.00 | Manager, p = 1.00 | Physician, missing data | Physiotherapist, p = 0.468; straight after game vs. 24 hrs after game: Athletictrainer, p = 1.00 | Manager, p = 1.00 | Physician, missing data | Physiotherapist, p = 0.891; 24 hrs before game vs. 24 hrs after game: Physician, p = 1.00). **(A-D)** Each dot represents one sample from one participant, samples from the same participant are connected by line across physical activity groups, significant changes (p-values) were tested using a linear mixed effect model with chronological age, timepoint (before game, after game or rested) and batch number as fixed and player id as random effect. Plots show median (bold line) with interquartile range (box) and 1.5-fold interquartile range (whiskers). Significance levels are indicated by * (p <= 0.05), ** (p <= 0.01) and NS. (p > 0.05). Cal.: GrimAge2 and FitAge predictions were calibrated to the actual age range of players.


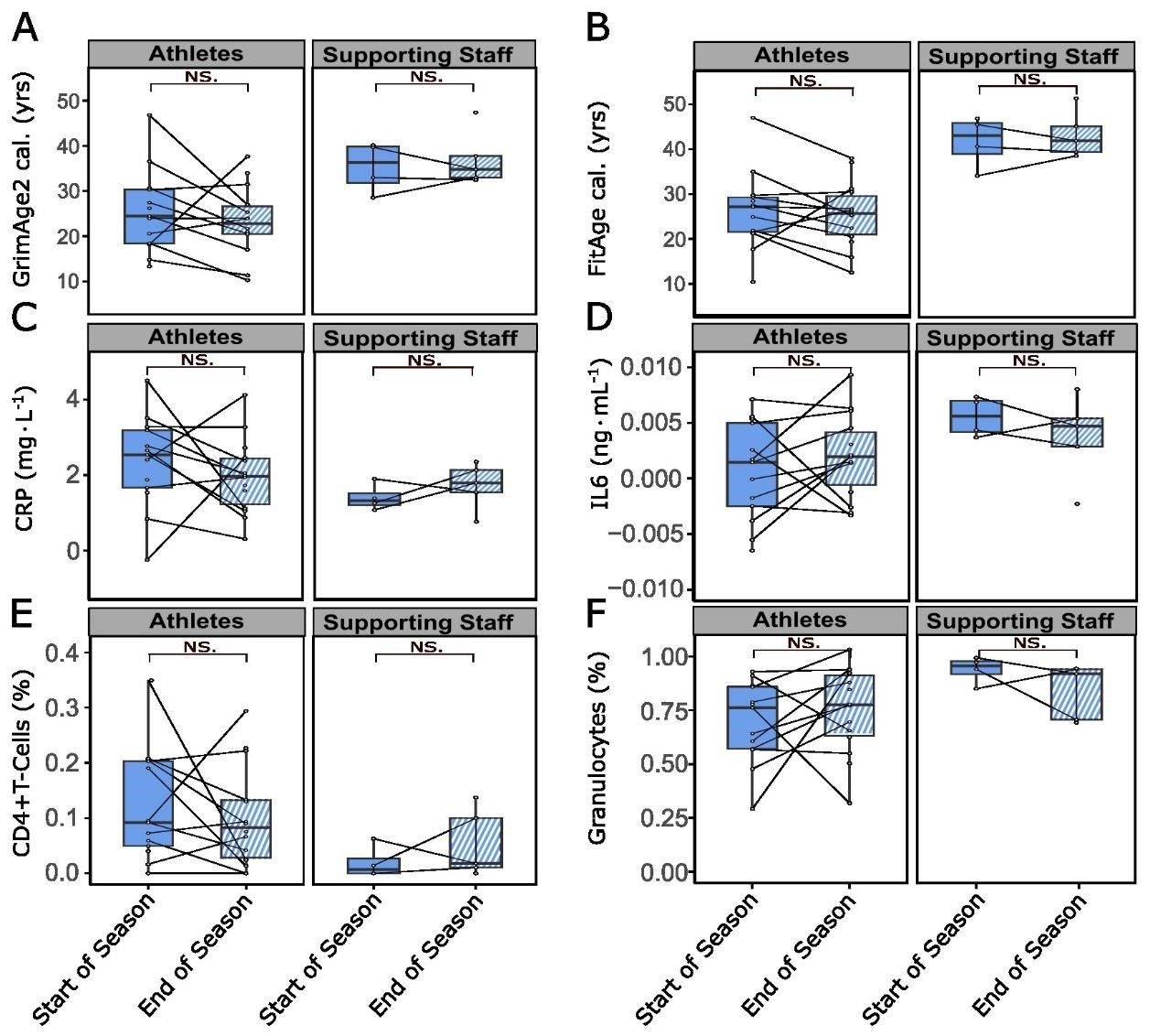


**Supplementary Figure 6: Long term effects of high physical load on DNAm-based age predictors and DNAm-derived immunological factors. (A-B)** Epigenetic profiles (DNAm) of saliva samples from athletes, collected during the beginning (samples 2, blue) and end of the season (sample 9, blue lined) from athlete participants (n < 19) and supporting staff as control (n = 4). The data was used to estimate DNAm-based biological aging clocks **(A) GrimAge2 cal.** (Athletes: beginning of season vs. end of season β = -3.24, 95% CI: [-8.75, 2.27], p = 0.219, f² = 0.082; Supporting Staff: beginning of season vs. end of season β = 1.74, 95% CI: [-5.89, 9.37], p = 0.673, f² = 0.024) and **(B) FitAge cal.** (Athletes: beginning of season vs. end of season β = -1.97, 95% CI: [-5.76, 1.82], p = 0.292, f² = 0.075; Supporting Staff: beginning of season vs. end of season β = 1.49, 95% CI: [-5.56, 8.53], p = 0.673, f² = 0.021). **(C-D)** Analysis of DNAm-based plasma protein surrogate factors **(C) Methylation-based estimator of CRP** (Athletes: beginning of season vs. end of season β = -0.42, 95% CI: [-1.34, 0.51], p = 0.338, f² = 0.060; Supporting Staff: beginning of season vs. end of season β = 0.31, 95% CI: [-0.36, 0.98], p = 0.400, f² = 0.101) and **(D) Methylation-based estimator of IL-6** (Athletes: beginning of season vs. end of season β = 0.0015, 95% CI: [-0.0017, 0.0049], p = 0.319, f² = 0.102; Supporting Staff: beginning of season vs. end of season β = -0.0011, 95% CI: [-0.0059, 0.0022], p = 0.592, f² = 0.030) and **(E-F)** immune cell type estimated proportion for **(E) CD4+ T-Cells** (Athletes: beginning of season vs. end of season β = -0.027, 95% CI: [-0.0951, 0.0416], p = 0.461, f² = 0.135; Supporting Staff: beginning of season vs. end of season β = 0.034, 95% CI: [-0.031, 0.099], p = 0.349, f² = 0.126) and **(F) Granulocytes** (Athletes: beginning of season vs. end of season β = 0.062, 95% CI: [-0.0961, 0.2192], p = 0.410, f² = 0.106; Supporting Staff: beginning of season vs. end of season β = -0.099, 95% CI: [-0.236, 0.039], p = 0.208, f² = 0.240). **(A-F)** Each dot represents one sample from one participant, samples from the same participant are connected by a line across physical activity groups, significant changes (p-values) were tested using a linear mixed-effect model with chronological age, timepoint (beginning of season, end of season), and batch number as fixed and player ID as a random effect. Plots show median (bold line) with interquartile range (box) and 1.5-fold interquartile range (whiskers). Significance levels are indicated by * (p ≤ 0.05), ** (p ≤ 0.01) and NS (p > 0.05). Cal.: GrimAge2 and FitAge predictions were calibrated to the actual age range of players.


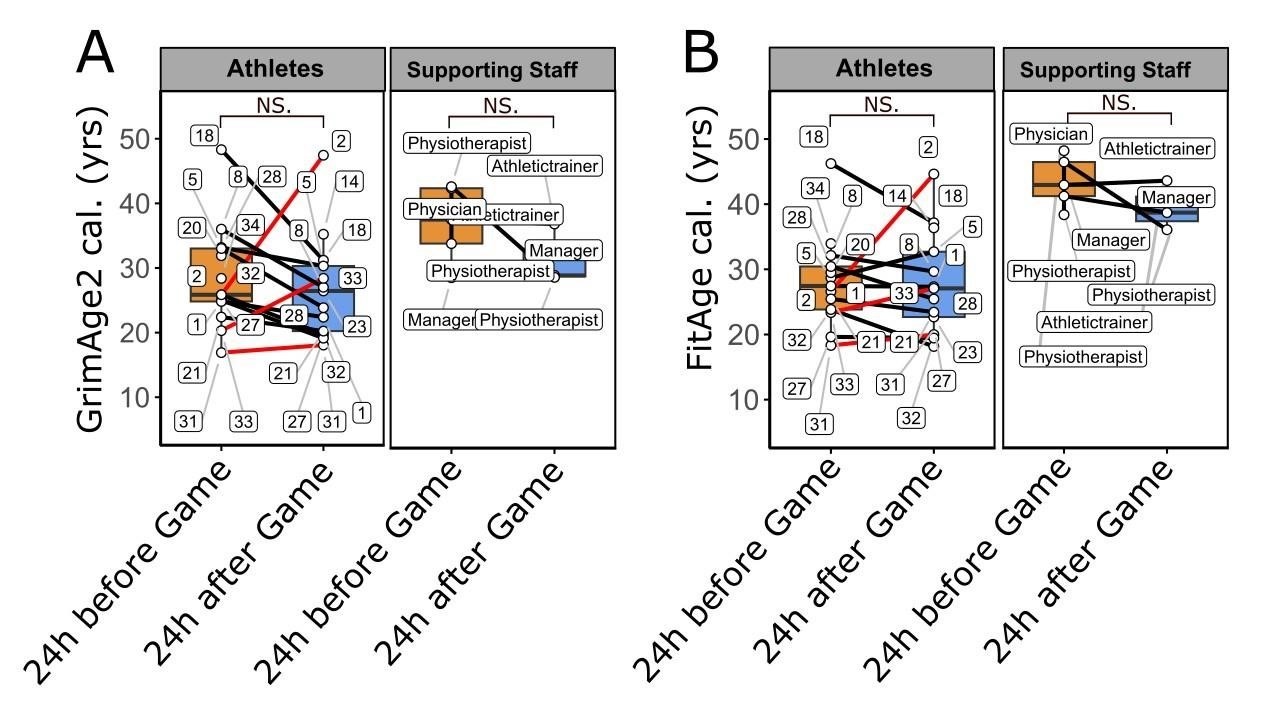


**Supplementary Figure 7:** **Short term effect of high physical load on DNAm-based age predictors.** **(A-B)** Epigenetic profiles (DNAm) of saliva samples from athletes (n < 19), collected during mid-season games (samples 3 and 5) 24 hrs before (before match: sample 3) and 24 h after (after match: sample 5) match day, were analyzed in addition to control samples from supporting staff members (n = 4). The data was used to estimate DNAm-based biological aging clocks **(A) GrimAge2 cal.** (Athletes: 24hrs before game vs. 24hrs after game, p = 0.77381, β = -0.8038, 95% CI: [-6.0849405, 4.477268], f² = 0.4642673; Supporting Staff: 24hrs before game vs. 24hrs after game, p = 0.266, β = -5.286, 95% CI: [-13.228274, 2.273042], f² = 0.2430808) and **(B) FitAge cal.** (Athletes: 24hrs before game vs. 24hrs after game, p = 0.730756, β = -0.6981, 95% CI: [-4.751307, 3.2114402], f² = 1.078346; Supporting Staff: 24hrs before game vs. 24hrs after game, p = 0.212, β = -3.983, 95% CI: [-9.471662, 1.506540], f² = 0.2781082) for each group. Each dot represents one sample from one participant, samples from the same participant are connected by line across physical activity groups, significant changes (p-values) were tested using a linear mixed effect model with chronological age, timepoint (24hrs before game vs. 24hrs after game) and batch number as fixed and player id as random effect. Plots show median (bold line) with interquartile range (box) and 1.5-fold interquartile range (whiskers). Significance levels are indicated by * (p <= 0.05), ** (p <= 0.01) and NS. (p > 0.05). Cal.: GrimAge2 and FitAge predictions were calibrated to the actual age range of players.

**Supplementary Table 1.** The Table shows direction of effect of 24 hrs before game vs. 24 hrs after game in **(A)** CK value change and **(B)** DNAmFitAge change. A table of the CK and DNamGrimAge2 status in group comparison shows the number of athlete participants suffering from an injury during season and having an increase or decrease in CK or DNAmGrimAge2 values between sample 3 and 5 with non-injury group as comparison (n = 11, as of 15 athletes only 11 had samples collected on both before and after).


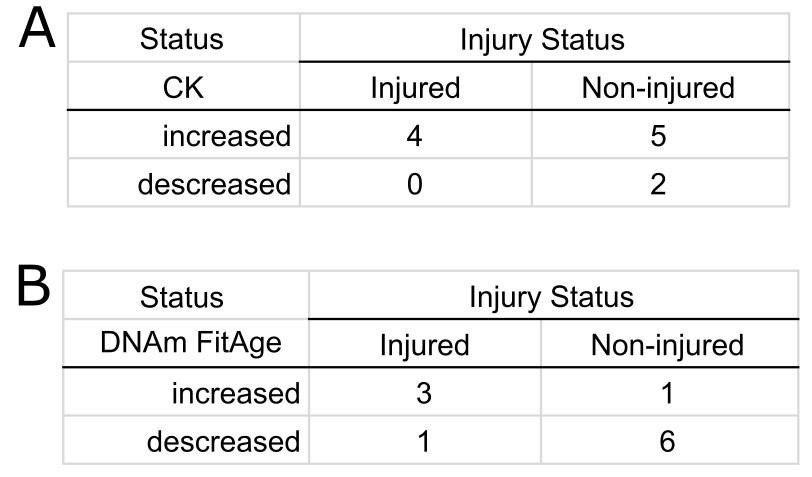

Supplement: Supplementary file 1 — Data S1. [file ACEL-24-e70182-s001.docx]
